# Large-Scale Modelling of the Environmentally-Driven Population Dynamics of Temperate *Aedes albopictus* (Skuse)

**DOI:** 10.1371/journal.pone.0149282

**Published:** 2016-02-12

**Authors:** Kamil Erguler, Stephanie E. Smith-Unna, Joanna Waldock, Yiannis Proestos, George K. Christophides, Jos Lelieveld, Paul E. Parham

**Affiliations:** 1 Energy, Environment and Water Research Center, The Cyprus Institute, 2121 Aglantzia, Nicosia, Cyprus; 2 Department of Plant Sciences, University of Cambridge, Downing Street, Cambridge, CB2 3EA, United Kingdom; 3 Sainsbury Laboratory, University of Cambridge, Bateman Street, Cambridge, CB2 1LR, United Kingdom; 4 Department of Life Sciences, Faculty of Natural Sciences, Imperial College London, South Kensington Campus, London SW7 2AZ, United Kingdom; 5 Computation-based Science and Technology Research Center, The Cyprus Institute, 2121 Aglantzia, Nicosia, Cyprus; 6 Department of Atmospheric Chemistry, Max Planck Institute for Chemistry, D-55128 Mainz, Germany; 7 Department of Public Health and Policy, Faculty of Health and Life Sciences, University of Liverpool, Liverpool L69 3GL, United Kingdom; 8 Grantham Institute for Climate Change, Department of Infectious Disease Epidemiology, School of Public Health, Faculty of Medicine, St. Mary’s campus, Imperial College London, London W2 1PG, United Kingdom; Universidade Federal do Rio de Janeiro, BRAZIL

## Abstract

The Asian tiger mosquito, *Aedes albopictus*, is a highly invasive vector species. It is a proven vector of dengue and chikungunya viruses, with the potential to host a further 24 arboviruses. It has recently expanded its geographical range, threatening many countries in the Middle East, Mediterranean, Europe and North America. Here, we investigate the theoretical limitations of its range expansion by developing an environmentally-driven mathematical model of its population dynamics. We focus on the temperate strain of *Ae. albopictus* and compile a comprehensive literature-based database of physiological parameters. As a novel approach, we link its population dynamics to globally-available environmental datasets by performing inference on all parameters. We adopt a Bayesian approach using experimental data as prior knowledge and the surveillance dataset of Emilia-Romagna, Italy, as evidence. The model accounts for temperature, precipitation, human population density and photoperiod as the main environmental drivers, and, in addition, incorporates the mechanism of diapause and a simple breeding site model. The model demonstrates high predictive skill over the reference region and beyond, confirming most of the current reports of vector presence in Europe. One of the main hypotheses derived from the model is the survival of *Ae. albopictus* populations through harsh winter conditions. The model, constrained by the environmental datasets, requires that either diapausing eggs or adult vectors have increased cold resistance. The model also suggests that temperature and photoperiod control diapause initiation and termination differentially. We demonstrate that it is possible to account for unobserved properties and constraints, such as differences between laboratory and field conditions, to derive reliable inferences on the environmental dependence of *Ae. albopictus* populations.

## Introduction

The tiger mosquito, *Aedes albopictus*, is an invasive species which poses health risks to humans through its capacity to act as a disease vector. Since the 1980s, *Ae. albopictus* has been the fastest spreading invasive animal species in the world [1]. Originally described by Skuse in 1894, it has been native to the Indian subcontinent and Southeast Asia [2], but has recently spread to all continents except Antarctica [3, 4], out-competing and displacing other native mosquito species [5–9].

*Ae. albopictus* demonstrates high flexibility in host biting preference; however, it has a strong preference for humans, making it an effective human arbovirus vector [10–13]. Its contribution to transmission of dengue (DENV) and chikungunya (CHIKV) viruses has been demonstrated in the field, while its capacity to transmit around 24 additional arboviruses under laboratory conditions has been also revealed [14, 15]. It has been the primary vector for CHIKV outbreaks in the Indian Ocean region from 2005 to 2006, with over three million cases reported [16]. The first occurrence of *Ae. albopictus* in Europe was reported in Albania in 1979 [17], and in 2007 it caused a CHIKV epidemic in Italy, where over 200 cases were reported [18, 19].

*Ae. albopictus* has the ability to reproduce in natural cavities such as tree or rock holes, and is rapidly adapting to domestic conditions [20]. Females can lay eggs in a wide variety of man-made containers, which has enabled the exploitation of international transit systems, increasing its invasive success and making humans partially responsible for its global translocation. To date, international used tire and lucky bamboo trades have been identified as the main means of *Ae. albopictus* transportation in temperate countries [2, 21].

In addition to its ecological plasticity, the temperate strain of *Ae. albopictus*, in contrast to the ancestral tropical strain, has developed the ability to lay diapausing eggs [22–24]. These eggs maintain a controlled dormant state, and have greatly improved reproductive success in colder climate zones by increasing survival during harsh winter conditions [25–27]. Furthermore, the poleward spreading of *Ae. albopictus* has been suggested to profit from climate change [28].

Understanding the dynamics and environmental dependency of vector-borne diseases is of paramount importance for planning effective management strategies and minimising health impacts of future disease outbreaks. Numerous research efforts have thus far focused on the population dynamics of vectors and vector-borne diseases. Lunde and coworkers have recently reviewed a range of models linking temperature to adult survival for the Afrotropical malaria vector *Anopheles gambiae*[29], and developed a combined *An. gambiae* and *An. arabiensis* (*An. gambiae* sibling species) population dynamics model [30]. Although a range of models have been developed for *Aedes aegypti* since 1993 [31–34], until recently, population models for *Ae. albopictus* did not exist. Nevertheless, many of the recent *Ae. albopictus* models exclude explicit references to environmental conditions [35–40].

One of the most notable environmentally-driven *Ae. albopictus* models has been developed by Erickson and coworkers in 2010 [41] and applied to predicting the suitability of the region of Texas, USA, to dengue fever [42]. To the best of our knowledge, the current state-of-the-art is represented by Tran *et al*. in 2013 [43] with a rainfall- and temperature-driven abundance model specifically calibrated for the French Riviera, France.

Many of the environmentally-driven population dynamics models share a common limitation. Although detailed accounts of carefully-planned laboratory experiments on key life-history traits exists, such as survival and development, parameters derived solely from these data are not applicable to a larger domain. The main difficulty is to measure micro-environmental conditions in potential habitats with an accuracy matching laboratory conditions.

Here, we aim to model *Ae. albopictus* population dynamics on a global scale. We adopt a novel strategy where we infer new parameters, based on laboratory and surveillance data, which would be appropriate for use with globally available gridded environmental variables. We regard each grid point, defined by environmental datasets, as a potential habitat, which span a large area—typically between 25 and 50 km^2^ along the equator.

We create a comprehensive database of environmental dependency extracted from the literature, and construct a model of a typical breeding site, and a model of the mechanism of diapause. We develop a deterministic stage- and age-structured population dynamics model incorporating these processes and several other environmentally-driven parameters. We perform Bayesian inference over the time-resolved high-quality surveillance data from the Emilia-Romagna region of Italy [44], and employ the laboratory-derived parameters as prior knowledge. We demonstrate the predictive capacity of the model over the region of inference, and argue that the approach makes modelling at large scales possible by comparing model predictions with regional surveillance reports throughout Europe.

## Methods

### Environmental dependency

Large-scale meta-analyses require combining data, often troubled by missing observations, obtained under various experimental configurations. Here, we combine data from sets of related laboratory studies to develop functional relationships between certain life-history traits and associated environmental variables. These relationships expressed in mathematical forms reflect expert opinion, and will be incorporated into the population dynamics model (sec. Population dynamics model).

In S1 Table, we present a complete list of model parameters and environmentally-driven life-history traits, along with fitted functional forms, data and references. As an overview, we studied daily survival probabilities, development rates and fecundity over a wide range of environmental conditions, and identified temperature as a key driver. Rainfall and human activities influence *Ae. albopictus* populations mainly by changing the properties of potential breeding sites. Data on the effects of relative humidity (RH) were limited, with an added difficulty of the strong temperature dependence of RH.

We interpolated missing observations by fitting appropriate functional forms to the data. We chose second degree polynomials for development times, fecundity and the time from emergence to first blood meal. We described diapausing egg survival with a linear equation, and the daily survival of non-diapausing eggs, larvae, pupae and adults with two-sided sigmoidal functions. We found that these were appropriate for delineating temperature boundaries on survival. Data were particularly limited for adult survival in extreme temperatures. For this, we assumed a lower limit of −3°C [45] and a higher limit of 37.5°C [46] with a standard deviation of 1°C in each case.

We defined development, *d*_1…3_, and survival of immature stages, *p*_1…3_, as functions of water temperature, *T*_*w*_, and defined adult survival, *p*_4_, and female fecundity, *F*_4_, as functions of air temperature, *T*_*a*_. We assumed a linear relationship between *T*_*a*_ and *T*_*w*_ where *T*_*w*_ = *T*_*a*_ − *Δ*_*T*_. The gridded environmental datasets often cover a large area and comprise daily averages (see section Environmental data); therefore, prior to calibration with surveillance data, we assumed no difference between *T*_*a*_ and *T*_*w*_, and set *Δ*_*T*_ = 0 with a standard deviation of 3°C. This assumption accounts for the previous assumptions of *Δ*_*t*_, which have been around 3–6°C [47–49].

Temperature-dependent development times for eggs, larvae and pupae, and temperature-dependent time to first blood meal for nulliparous females were reported under roughly comparable experimental conditions. We derived temperature-dependent daily survival of adults from the reports of daily mortality and average adult life expectancy assuming fixed daily mortality. Temperature-dependent survival of eggs, larvae and pupae were reported mostly in terms of total survival over the duration of each life stage. In order to calculate daily survival, we estimated the duration of each stage from the best-fitting curves, and assumed fixed daily mortality under relatively stable environmental conditions. We estimated temperature-dependent fecundity from the reports of total eggs per female in a lifetime. Accordingly, we estimated lifespan from the best-fitting curves of adult survival, and calculated the number of eggs laid daily. We reserve the discussion of density-dependence and diapause to the following sections due to their complexity.

For each group of data and associated functional forms, we performed curve-fitting using non-linear least-squares regression. We defined multivariate Gaussian distributions for the parameter set of each functional form. These distributions represents our best estimates of possible values and variabilities in these parameters. In Bayesian terms, these are called prior distributions as they assign probabilities for parameter values prior to comparing model output with observed surveillance data. For certain parameters for which explicit experimental data was not available, we assigned appropriate distributions and boundaries and relied on the inference process to arrive at the best estimates.

### Model of the breeding site

We begin describing the model by first developing a model for the habitat where *Ae. albopictus* populations are presumed to live and breed. In the scope of this work, we assume that a specific vector population is confined to an isolated territory of a predefined size. Given the availability and type of surveillance data, such a territory could be as small as the locality of a trap or as large as the area represented by a grid cell of an environmental dataset. Since we used surveillance data to calibrate model parameters, the model became adapted to describing populations restricted to the habitat represented by the surveillance data. As *Ae. albopictus* tend to have a short flight range within several hundred meters and stay close to the ground [20], we assumed that the effect of migration is negligible for an area this large.

Within such a territory, which we refer to as a breeding site, we expect to have various containers, cavities or similar concave structures capable of hosting immature stages of the vector. We assumed that such breeders are randomly distributed throughout the breeding site, and, for the sake of simplicity, are similar in structure and habitability.

Whether a breeder is habitable or not depends on many environmental factors, including the surface area of the water it holds and nutrients it provides for larvae and pupae. Here, we used the term carrying capacity as a relative measure of breeder habitability.

Rainfall has a strong influence on carrying capacity; however, unlike the ancestral strains living in tree-holes, modern *Ae. albopictus* often breed in human-made water sources independent of rainfall [15]. Prompted by this, we modelled daily changes in carrying capacity, throughout a breeding site, as a function of daily average precipitation and human population density:

$$\begin{array}{c} B_{t+1}=\alpha_{pdens}\;p_{dens}+\alpha_{dprec}\;d_{prec_{t}}+\alpha_{evap}\;B_{t}, \end{array}$$ (1)

where $B_{t}$ represents carrying capacity on day *t*, *p*_*dens*_ is the human population density, and *d*_*prec*_*t*__ is the average precipitation on day *t*. The scaling factors *α*_*pdens*_ and *α*_*dprec*_ weigh relative contributions of population density and precipitation, respectively. Finally, *α*_*evap*_ is the ratio of the capacity retained daily despite evaporation and absorption. Although it is possible to accommodate the effect of changing human activity, due to limitations in environmental datasets and the availability of data for validation, here, we incorporated a fixed human influence as a first approximation.

Eq 1 is equivalent to the unscaled version of Eq 10 in White *et al*.[50] and Eq 6 in Christiansen-Jucht *et al*.[51] where only daily average precipitation is used as a factor (see S1 Text for a derivation). The scaled version calculates average, not aggregate, effects of recent rainfall events, and, thus, limits the amount of capacity transferrable to the subsequent day. We argue that the scaled version is more realistic given the limited availability of breeders in a breeding site. As a result, we modelled carrying capacity in terms of average precipitation and human activities weighed by an exponential distribution with mean −1/ln *α*_*evap*_ days,

$$\begin{array}{c} K_{t}=\frac{1-\alpha_{evap}}{1-\alpha_{evap}^{t}}\;B_{t}. \end{array}$$ (2)

This is a simple, yet generic, model capable of accounting for a range of environmental variables influencing a typical breeding site. It can be applied to different habitats and climatic conditions as an informative first approximation. It can, of course, be elaborated upon and tailored for a specific region given data availability.

### Model of diapause

Adult females of temperate *Ae. albopictus* are able to lay diapausing eggs which could preserve their state as pharate first instar larvae. Diapausing and non-diapausing eggs have similar abilities to withstand the cold—Thomas and coworkers reported in 2012 a difference of only 1 or 2°C in their lowest temperature of survival [52]. However, the main advantage of diapausing seems to protect the vulnerable life stages, such as larva and pupa, against harsh winter conditions [53].

Literature on the precise nature and factors regulating this behaviour is limited, however, low temperature and short day length, *i.e*. photoperiod, have been associated with the induction of egg diapause [20, 53–55]. The photoperiod threshold below which diapause is induced in 50% of a population is called the critical photoperiod (CPP) [56]. Diapausing eggs produced in such conditions depend mainly on photoperiod and temperature to hatch—Mori and coworkers showed in 1981 that an increase in temperature alone would not be sufficient to trigger hatching [53].

In order to model this behaviour, we considered two seasons, a favourable and an unfavourable season, delineated by the entry to and exit from diapause. In the favourable season, an adult female *Ae. albopictus* lays normal eggs, which will hatch and follow up the developmental stages as expected. In the unfavourable season, however, she produces diapausing eggs, which will cease development until the arrival of the favourable season.

To mark the beginning of the unfavourable season, we imposed a threshold on both temperature, *T*_crt_, and photoperiod, CPP. We assumed that neither of these factors alone will trigger diapause—a case which results in the production of normal, non-diapausing, eggs. When the days are short and the temperature is low, females begin to produce eggs to become diapausing eggs. We assumed that these are initially identical to non-diapausing eggs, but they refrain from hatching, and become diapausing, at the end of their development. To keep track of these eggs in the model, we referred to them as tagged eggs, Egg*, separately from diapausing (Egg°) and non-diapausing (Egg) eggs.

We further assumed that females would not suddenly switch from producing normal eggs to tagged eggs, but increasingly more eggs would be tagged and, therefore, destined to diapause. The daily production of tagged eggs per adult female can be defined as the daily average number of eggs laid by a female (fecundity), *F*_4_, multiplied by the fraction of tagged eggs, *p*_dp_. In essence,

$$\begin{array}{c} F_{4}\times p_{\text{dp}},\;\text{where}\;p_{\text{dp}}=\min\left\{1,\left(1+t-t_{\text{dp}}\right)p_{s}\right\}. \end{array}$$

In this equation, *t*_dp_ corresponds to the day the unfavourable season begins and *p*_*s*_ represents the initial fraction of tagged eggs. According to this, the fraction of tagged eggs laid on day *t*_dp_ is *p*_*s*_, and this increases by *p*_*s*_ every day until only tagged eggs are laid, *i.e*. until *p*_dp_ reaches unity.

We also employed a gradual transformation for hatching. We assumed that, once the day length and temperature both increase above their respective thresholds, the daily fraction of diapausing eggs entering developmental cycle is *p*_*n*_. As a result, instead of forcing a sharp synchronised switch between the states of diapause and non-diapause, we enabled gradual transformations, and let the rates be enforced by data.

### Population dynamics model

We developed a deterministic stage- and age-structured discrete-time model for the population dynamics of *Ae. albopictus*. The model is influenced by the environmentally-driven population dynamics model of *An. gambiae*, which was developed in Parham *et al*. 2012 [49]. The original model successfully compiles a variety of environmental dependencies in a stage-structured, discrete-time model with daily time-steps. Daily time-steps enable a straightforward interpretation of experimental work and field observations collected on daily basis.

While retaining the original framework, we substantially revised the model to describe the four major developmental stages, eggs, larvae, pupae and adults, while employing a more realistic age-structured developmental process. We modelled temperature-dependence of survival, development rate and female fecundity, and also accounted for the diapausing behaviour and environmental-dependence of the carrying capacity.

The revised set of equations comprises a total of 48 parameters and 7 dynamically changing variables. We implemented the model in ANSI C and developed a Python (v2.7) interface for running it. The code and related material are available as a Python package called albopictus, which is given in S1 File. Installation and usage instructions are given in S2 Text.

The original model of Parham and coworkers employed a projection matrix for three types of life-events: fecundity, survival and development [49]. Daily average values of these events were estimated to calculate the state of the population in the subsequent day. Here, we adopted a similar approach, but, treated development separately by introducing an age-structured developmental process for the immature stages. In addition to the different egg stages described above, we included larvae (Larva), pupae (Pupa), naive females (Naive: nulliparous adult females that have yet to acquire a blood meal) and parous adult females (Adult) in the model.

**Fig 1:**
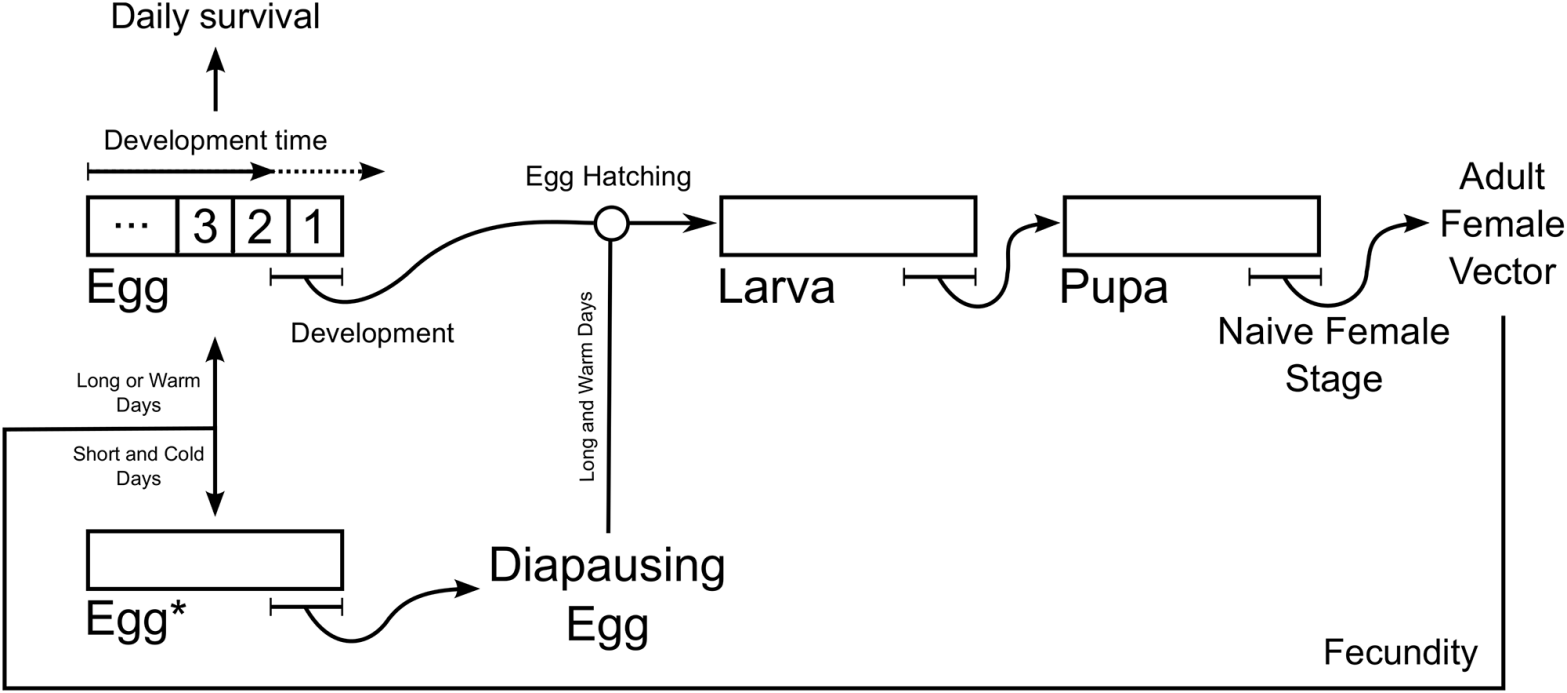
The stage- and age-structured time-difference equations model of *Ae. albopictus*.

The resulting model of *Ae. albopictus* life-cycle can be summarised as

$$\begin{array}{c} n_{t+1}=G\left(n,t\right)+M\left(n,t\right)\times n_{t}, \end{array}$$ (3)

where $n_{t}=\left({\text{Egg}}_{t},{\text{Egg}}_{t}^{*},{\text{Egg}}_{t}^{0},{\text{Larva}}_{t},{\text{Pupa}}_{t},{\text{Naive}}_{t},{\text{Adult}}_{t}\right)$, **M**(**n**, *t*) is the projection matrix, and the vector **G**(**n**, *t*) represents the developmental processes. A graphical representation of the model can be seen in Fig 1.

#### Modelling development

We assumed that under fixed environmental conditions, development time for an immature stage is fixed. For instance, if the expected duration of development of a non-diapausing egg is *d* days, 1/*d* of this process will be completed each day for a batch of eggs laid on the same day. After *d* days, the number of eggs completing their development, Egg_dev_, will be

$$\begin{array}{c} {\text{Egg}}_{\text{dev}}={\text{Egg}}_{0}\times p^{d}, \end{array}$$

where Egg_0_ is the number of eggs at the beginning and *p* is daily survival probability. Under variable environmental conditions with fixed development time, this equation becomes

$$\begin{array}{c} {\text{Egg}}_{\text{dev}}={\text{Egg}}_{0}\prod_{t=1}^{d}p_{t}, \end{array}$$

where *p*_*t*_ is the daily survival for day *t*. However, the development time is also variable from day to day under changing environmental conditions. Hence, the total development time, *d*_total_, can be calculated in terms of the minimum number of days required for the sum of daily fractions of development, 1/*d*_*t*_, to exceed 1,

$$\begin{array}{c} d_{\text{total}}=\inf\left\{n\in N^{+}:\sum_{t=1}^{n}1/d_{t}\geq 1\right\}, \end{array}$$

where inf stands for infimum, *i.e*. the greatest lower bound, and *N*^+^ represents the set of positive integers. Replacing *d* with *d*_total_, we can write Egg_dev_ as

$$\begin{array}{c} {\text{Egg}}_{\text{dev}}={\text{Egg}}_{0}\prod_{t=1}^{d_{\text{total}}}p_{t}. \end{array}$$

According to this process, no eggs are allowed to progress onto the subsequent stage until *d*_total_ days have passed, which ensures that eggs develop in a delayed fashion with regards to their daily development rates and survival probabilities.

We implemented a similar process for all types of normal eggs, larvae, pupae and naive females, and defined **G**(**n**, *t*) as

$$\begin{array}{c} G\left(n,t\right)=\left[\begin{array}{c} -{\text{Egg}}_{\text{dev}}\\ -{\text{Egg}}_{\text{dev}}^{*}\\ {\text{Egg}}_{\text{dev}}^{*}\\ {\text{Egg}}_{\text{dev}}-{\text{Larva}}_{\text{dev}}\\ {\text{Larva}}_{\text{dev}}-{\text{Pupa}}_{\text{dev}}\\ \frac{1}{2}{\text{Pupa}}_{\text{dev}}-{\text{Naive}}_{\text{dev}}\\ {\text{Naive}}_{\text{dev}} \end{array}\right]. \end{array}$$ (4)

According to this equation, upon completing their development, tagged eggs become diapausing, non-diapausing eggs become larvae, larvae become pupae, half of pupae become naive females and these, in turn, become adults.

Here, we exclusively considered adult females as only they obtain blood meal and pose a threat to humans. We assume a sex ratio of 1:1 [46] and simplify different phases of the air-borne stage to nulliparous and parous females. We assumed that once sufficient time to first blood meal passes, *i.e*. the development time for naive females, *t*_*bm*_, the adult stage commences. We assumed that, during the adult stage, survival is independent of age, egg laying is daily and females have regular access to males and blood meals.

We note here that although we employed the assumption of age-independent mortality for all life-stages, the developmental process as described allows one to relax this assumption in a straightforward manner for future enhancements of the model.

We also note that observing the effects of carrying capacity on *Ae. albopictus* populations is not straightforward, mainly because there is not a standard method of calculating it for different experimental setups. Instead, contributing factors are often reported. For instance, many studies suggest a trend of increase in development times for immature stages as a result of increased vector density or decreased food ration [57–60].

Here, we focus on two studies where the authors reported combined effects of multiple factors. Briegel *et al*. in 2001 investigated the effects of an increase in larval density and temperature on development times [58]. Gavotte *et al*. in 2009 reported a density-dependent increase in development times and decrease in the survival of immature stages in a single study [60]. Although these results are not directly comparable, because carrying capacity is a limiting factor in both of them, it is possible to use them to incorporate temperature-dependence into a density-dependent development model.

In order to do this, we defined a temperature-dependent polynomial function,

$$\begin{array}{c} \tau \left(T_{w}\right)=\alpha_{a}+\alpha_{b}\;T_{w}+\alpha_{c}\;T_{w}^{2}, \end{array}$$

with three parameters, *α*_*a*⋯*c*_, which describes the increase in development times as density increases as reported in Briegel *et al*., 2001. In addition, we defined a power function to model the density-dependent increase in development times at 28°C based on the data from Gavotte *et al*., 2009.

As a consequence, we hypothesised that the combined effect of density and temperature can be written as

$$\begin{array}{c} \begin{array}{ccc} d_{23}\left(\mu_{t},T_{w}\right) & = & \alpha_{d}\;\mu_{t}^{\alpha_{e}\;\tau \left(T_{w}\right)}, \end{array} \end{array}$$

with two parameters, *α*_*d*_ and *α*_*e*_, where *d*_23_(*μ*_*t*_, *T*_*w*_) represents the fractional change in both *d*_2_ and *d*_3_, and *μ*_*t*_ represents the density of immature stages at time *t* as reported in Gavotte *et al*., 2009. By assuming that container volume is proportional to carrying capacity, K, we defined $\mu_{t}=\left({\text{Larva}}_{t}+{\text{Pupa}}_{t}\right)/K_{t}$. For studies where density is not a limiting factor, we assumed *μ* ≈ 0, and enforced the minimum value of *d*_23_(*μ*_*t*_, *T*_*w*_) to be 1, meaning that in these cases, development times are determined only by temperature, *i.e*. *d*_2_(*T*_*w*_) and *d*_3_(*T*_*w*_). As in the previous section, we defined a prior distribution for density-related parameters by performing curve-fitting with non-linear least-squares regression (see S1 Table). In S1 Fig, we present the agreement between observed and predicted development times.

#### Modelling survival

In addition to development, we modelled survival and fecundity using a projection matrix. We assumed that each day a fraction of each life stage survives and a new batch of eggs are laid. We described survival probabilities for diapausing eggs with *p*_0_, tagged and non-diapausing eggs with *p*_1_, larvae with *p*_2_, pupae with *p*_3_, and naives and adults with *p*_4_. We also described daily average fecundity with *F*_4_.

We considered density-dependent survival of immature stages and represented this with *p*_*LD*_. In the absence of detailed records, we assumed that the effects of density and temperature on survival are independent resulting in *p*_2_ = *p*_2_(*T*_*w*_)*p*_*LD*_ and *p*_3_ = *p*_3_(*T*_*w*_)*p*_*LD*_.

In summary, the dependence of survival, development times, fecundity and time to first blood meal can be listed as follows.

$$\begin{array}{c} \begin{array}{cccccc} p_{0} & = & p_{0}\left(T_{a}\right) & F_{4} & = & F_{4}\left(T_{a}\right)\\ p_{1} & = & p_{1}\left(T_{w}\right) & d_{1} & = & d_{1}\left(T_{w}\right)\\ p_{2} & = & p_{2}\left(T_{w}\right)\;p_{LD}\left(\mu_{t}\right) & d_{2} & = & d_{2}\left(T_{w}\right)\;d_{23}\left(\mu_{t},T_{w}\right)\\ p_{3} & = & p_{3}\left(T_{w}\right)\;p_{LD}\left(\mu_{t}\right) & d_{3} & = & d_{3}\left(T_{w}\right)\;d_{23}\left(\mu_{t},T_{w}\right)\\ p_{4} & = & p_{4}\left(T_{a}\right) & t_{bm} & = & t_{bm}\left(T_{a}\right). \end{array} \end{array}$$

The projection matrix, **M**(**n**, *t*), describing these processes can be written as

$$\begin{array}{c} M\left(n,t\right)=\left[\begin{array}{ccccccc} p_{1} & 0 & 0 & 0 & 0 & 0 & \left(1-I_{\text{unfav}}p_{\text{dp}}\right)\;F_{4}\\ 0 & p_{1} & 0 & 0 & 0 & 0 & I_{\text{unfav}}\;p_{\text{dp}}\;F_{4}\\ 0 & 0 & I_{\text{fav}}\;\left(1-p_{n}\right) & 0 & 0 & 0 & 0\\ 0 & 0 & I_{\text{fav}}p_{n} & p_{2} & 0 & 0 & 0\\ 0 & 0 & 0 & 0 & p_{3} & 0 & 0\\ 0 & 0 & 0 & 0 & 0 & p_{4} & 0\\ 0 & 0 & 0 & 0 & 0 & 0 & p_{4} \end{array}\right], \end{array}$$

where $I_{\text{fav}}$ and $I_{\text{unfav}}$ are indicator functions signalling the initiation and termination of diapause

$$\begin{array}{c} I_{\text{fav}}=\left\{\begin{array}{cc} 1 & \text{if}\;T_{a}\geq T_{\text{crt}}\;\text{and}\;\text{photoperiod}\geq \text{CPP}\\ 0 & \text{otherwise} \end{array}\right. \end{array}$$

and

$$\begin{array}{c} I_{\text{unfav}}=\left\{\begin{array}{cc} 1 & \text{if}\;T_{a}<T_{\text{crt}}\;\text{and}\;\text{photoperiod}<\text{CPP}\\ 0 & \text{otherwise} \end{array}\right.. \end{array}$$

In the model, diapausing initiates only when both temperature and photoperiod fall below their respective thresholds. If one or both of them are above threshold only non-diapausing eggs are laid. Diapausing eggs hatch if and only if both temperature and photoperiod are above threshold. In S2 Fig, we present a diagram depicting the roles of $I_{\text{fav}}$ and $I_{\text{unfav}}$ in making this decision.

The resulting time-difference equations can be written as

$$\begin{array}{c} \begin{array}{ccccc} {\text{Egg}}_{t+1} & = & -{\text{Egg}}_{\text{dev}} & + & p_{1}{\text{Egg}}_{t}+\left(1-I_{\text{unfav}}p_{\text{dp}}\right)\;F_{4}\;{\text{Adult}}_{t}\\ {\text{Egg}}_{t+1}^{*} & = & -{\text{Egg}}_{\text{dev}}^{*} & + & p_{1}{\text{Egg}}_{t}^{*}+I_{\text{unfav}}\;p_{\text{dp}}\;F_{4}\;{\text{Adult}}_{t}\\ {\text{Egg}}_{t+1}^{0} & = & {\text{Egg}}_{\text{dev}}^{*} & + & I_{\text{fav}}\;\left(1-p_{n}\right)\;{\text{Egg}}_{t}^{0}\\ {\text{Larva}}_{t+1} & = & {\text{Egg}}_{\text{dev}}-{\text{Larva}}_{\text{dev}} & + & I_{\text{fav}}p_{n}\;{\text{Egg}}_{t}^{0}+p_{2}{\text{Larva}}_{t}\\ {\text{Pupa}}_{t+1} & = & {\text{Larva}}_{\text{dev}}+{\text{Pupa}}_{\text{dev}} & + & p_{3}{\text{Pupa}}_{t}\\ {\text{Naive}}_{t+1} & = & \frac{1}{2}{\text{Pupa}}_{\text{dev}}-{\text{Naive}}_{\text{dev}} & + & p_{4}{\text{Naive}}_{t}\\ {\text{Adult}}_{t+1} & = & {\text{Naive}}_{\text{dev}} & + & p_{4}{\text{Adult}}_{t}. \end{array} \end{array}$$

### Environmental data

We extracted daily average air temperature, 2 m above ground (t2m), and daily precipitation from the gridded climate dataset (E-OBS) of the EU-FP6 project ENSEMBLES [61]. This dataset has a resolution of 0.25°, corresponding to approximately 25 km between grid points along the equator. It covers Europe, the Mediterranean region and the north-west of the Middle East. We employed the UN-adjusted “Gridded Population of the World: Future Estimates” (GPWFE) dataset [62] with matching resolution for 2010 as the source of human population density. We also applied a land/water mask based on ISLSCP2 [63] selecting all grid points with more than 1% land coverage or with non-zero human population density for analysis.

We note that, due to the difficulty of distinguishing different types of precipitation in globally-available gridded environmental data sources, we treated all precipitation events as rainfall.

Finally, we used the “geosphere” package of R (v3.1.1) to calculate photoperiod for any given latitude and date.

### Parameter inference

We employed the *Ae. albopictus* surveillance data from the Public Health Service, Emilia-Romagna [44]. This includes summary statistics of biweekly ovitrap collections during May-October from 2008 to 2012, inclusive, covering 7 provinces of the region: Bologna, Ferrara, Modena, Piacenza, Parma, Ravenna and Reggio Emilia. Ovitraps were distributed exclusively over urbanised areas because of their relevance for public health risk assessment (see Fig 1 in Albieri *et al*. 2010 [64]). We chose this unique dataset for parameter inference because of the abundance, reliability and frequency of the collections.

In order to perform the inference, we adopted a scenario where *Ae. albopictus* is introduced to a region in winter with a certain number of diapausing eggs. We allowed the eggs to initiate and establish a vector population for a year, during 2007, and compared estimated and observed egg counts from 2008 until the end of 2012.

We assumed that the magnitude of the average egg count per ovitrap is distributed normally with mean *δ*_*r*,*ω*_ and standard deviation *σ*_*r*,*ω*_ for province *r*, where *ω* represents the two weeks prior to data collection. We employed the reported means and used standard error of the mean as an estimate of variability in the observed means. We also assumed a weak time-dependence where the difference between consecutive observations is distributed normally with mean *δ*_*r*,*ω*_ − *δ*_*r*,*ω*′_ and standard deviation $\sigma_{r,\omega }^{\prime }$, where *ω*′ indicates the previous observation. We assigned $\sigma_{r,\omega }^{\prime }$ the pooled standard error of the mean,

$$\begin{array}{c} \sigma_{r,\omega }^{\prime }=\sqrt{\left(\frac{1}{n_{r,\omega }}+\frac{1}{n_{r,\omega^{\prime }}}\right)\;\left(\frac{n_{r,\omega }\left(n_{r,\omega }-1\right)\sigma_{r,\omega }^{2}+n_{r,\omega^{\prime }}\left(n_{r,\omega^{\prime }}-1\right)\sigma_{r,\omega^{\prime }}^{2}}{n_{r,\omega }+n_{r,\omega^{\prime }}-2}\right)}, \end{array}$$

where *n*_*r*,*ω*_ is the number of ovitraps in province *r* during *ω*. For simplicity, we assumed that only consecutive observations are correlated, and grouped them for each year. As a consequence, the initial *ω* for each year corresponds to the second data point at the beginning of each surveillance period.

As a result, we defined the probability of observing on average *δ*_*r*,*ω*_ eggs per ovitrap for province *r* during *ω* as

$$\begin{array}{ll} \mathrm{Pr}(\delta |\theta )= & \underset{r}{\prod }\underset{\omega }{\prod }\frac{1}{\sigma_{r,\omega }\sqrt{2\pi }}\exp\left\{-\frac{1}{2}{\left(\frac{\delta_{r,\omega }-y\left(r,\omega ,\theta \right)}{\sigma_{r,\omega }}\right)}^{2}\right\}\times \\ & \underset{r}{\prod }\underset{\omega }{\prod }\frac{1}{\sigma_{r,\omega }^{\prime }\sqrt{2\pi }}\exp\left\{-\frac{1}{2}{\left(\frac{\left(\delta_{r,\omega }-\delta_{r,\omega^{\prime }}\right)-\left(y\left(r,\omega ,\theta \right)-y\left(r,\omega^{\prime },\theta \right)\right)}{\sigma_{r,\omega }^{\prime }}\right)}^{2}\right\}, \end{array}$$

where *y*(*r*, *ω*, *θ*) refers to simulated data for the corresponding data point performed with a parameter vector, *θ*, of size 48, which comprises all the parameters listed in S1 Table.

In order to generate simulated data, we selected a set of grid points for each province overlapping with the regions of high ovitrap density (see S3 Fig). For each grid point, we simulated the sum of all eggs laid, *i.e*. non-diapausing, tagged and diapausing eggs, during two weeks prior to each data collection. The average value corresponds to *y*(*r*, *ω*, *θ*) in comparison to the observation *δ*_*r*,*ω*_.

We performed Bayesian inference to arrive at a set of parameter vectors with high probabilities given the observations and prior knowledge. The posterior probability of a parameter vector, Pr(*θ*|*δ*), is proportional to the likelihood of simulating the data with the model and *θ*, Pr(*δ*|*θ*), and the prior probability assigned to *θ*, Pr(*θ*),

$$\begin{array}{c} \mathrm{Pr}(\theta |\delta )\propto \mathrm{Pr}(\delta |\theta )\;\mathrm{Pr}(\theta ). \end{array}$$

We used the adaptive basin-hopping Markov chain Monte Carlo (MCMC) algorithm, implemented in the hoppMCMC package of python (v2.7), to sample a set of high-probability parameter vectors from the posterior distribution [65]. The algorithm employs a variable acceptance probability to jump between two high-probability regions of a posterior distribution. The acceptance probability is defined as

$$\begin{array}{c} \mathrm{Pr}\left(\theta ,\theta^{\prime },T\right)=\min\left(1,\;\exp\left\{\frac{f\left(\theta \right)-f\left(\theta^{\prime }\right)}{T_{\text{ann}}}\right\}\right), \end{array}$$ (5)

where *f*(*θ*) is the objective function and *θ* and *θ*′ are current and proposed parameter vectors, respectively. The annealing temperature, *T*_ann_, is used to regulate tolerance similar to simulated annealing [66].

We employed an objective function which is half the square of the Mahalanobis distance between the data and simulations,

$$\begin{array}{c} f\left(\theta \right)=\frac{1}{2}{\left(x-\mu \right)}^{T}\Sigma^{-1}\left(x-\mu \right), \end{array}$$ (6)

where *μ* represents an expectation either for a parmeter value or a surveillance data point, *x* is the corresponding observation, *Σ* is the covariance matrix for all *μ* and ^*T*^ indicates matrix transpose. Since *f*(*θ*) is proportional to −lnPr(*θ*|*δ*), Eq 5 yields the Metropolis-Hastings acceptance criterion when *T*_ann_ = 1. In essence, when used with Eq 6, *T*_ann_ acts as a scaling factor for covariance.

Following the identification of a set of high-probability parameter vectors, we iterated independent MCMC chains with these vectors with a fixed annealing temperature, *T*_ann_ = 10, to sample 100 parameter vectors for each. Each of these 100 vectors belongs to a posterior mode, which will be discussed in the next section.

## Results and Discussion

### Evaluation of the inference over Emilia-Romagna

Since a change in parameter values results in a change in model output, as one moves further away from the proximity of a parameter setting, model behaviour can change dramatically, and this may result in bifurcations. A complex system with many parameters is likely to possess various types of behaviour each characterised by a different parameter setting. In this context, we define a posterior mode, Θ, to be a set of relatively higher-probability parameter values separated from their surroundings by a set of relatively lower-probability parameter values. Each posterior mode is associated with a similar behaviour, which may be unique to that mode or shared among different modes regardless of the degree of separation between them.

Using the Bayes’ theorem, we see that the marginal probability of a particular mode is proportional to its prior probability, *Pr*(Θ), multiplied by the expected value of the likelihood with respect to the prior,

$$\begin{array}{c} \mathrm{Pr}(\Theta |\delta )\propto \int \mathrm{Pr}(\delta |\theta ,\Theta )\mathrm{Pr}(\theta |\Theta )Pr(\Theta )d\theta . \end{array}$$

Since we do not set a prior preference for any posterior mode, Pr(Θ) is a constant. The prior probability, Pr(*θ*|Θ), and the likelihood of each parameter, Pr(*δ*|*θ*, Θ), regardless of which posterior mode *θ* belongs to, is as defined in the Methods (sec. Parameter inference). Given a set of posterior modes, S, and a set of parameters sampled from the modes, Pr(Θ|*δ*) can be estimated using importance sampling,

$$\begin{array}{c} \begin{array}{ccc} \mathrm{Pr}(\Theta |\delta ) & = & C\frac{1}{N_{\Theta }}\sum_{i=1}^{N_{\Theta }}\frac{\mathrm{Pr}(\delta |\theta ,\Theta )\mathrm{Pr}(\theta |\Theta )}{\mathrm{Pr}(\theta |S)}\\ & = & C^{\prime }\frac{1}{N_{\Theta }}\sum_{i=1}^{N_{\Theta }}\frac{\mathrm{Pr}(\theta |\delta ,\Theta )}{\mathrm{Pr}(\theta |S)}\\ & = & C^{\prime \prime }\frac{1}{N_{\Theta }}\sum_{i=1}^{N_{\Theta }}\frac{1}{\mathrm{Pr}(\theta |S)}e^{-f(\theta )}, \end{array} \end{array}$$

where *N*_Θ_ is the number of samples in Θ, *f*(*θ*) is the objective function (Eq 6) and Pr(θ|S) is the probability of sampling *θ* from S. In this equation, C, $C^{\prime }$ and $C^{\prime \prime }$, are normalising constants:

$$\begin{array}{c} C^{\prime \prime }=\frac{1}{\sum_{\Theta \in S}\frac{1}{N_{\Theta }}\sum_{i=1}^{N_{\Theta }}\frac{1}{\mathrm{Pr}(\theta |S)}e^{-f(\theta )}}. \end{array}$$

Since we used experimental data from the literature and environmental variables from gridded datasets, and modeled *Ae. albopictus* populations at a scale of about 25 km^2^ per grid location, it is unlikely to obtain a perfect match to both the surveillance data and the prior expectations. In order to overcome the numerical problems associated with low probabilities, we employed a scaling factor, *T*_tol_, for the standard deviation of the objective function,

$$\begin{array}{c} \mathrm{Pr}\left(\Theta |\delta ,T_{\text{tol}}\right)\propto \frac{1}{N_{\Theta }}\sum_{i=1}^{N_{\Theta }}\frac{1}{\mathrm{Pr}(\theta |S)}e^{-f\left(\theta \right)/T_{\text{tol}}}. \end{array}$$ (7)

Effectively, *T*_tol_ is comparable to the annealing temperature, *T*_ann_, in Eq 5, as it regulates tolerance among the set of posterior modes. The ratio of the probabilities of two posterior modes when *T*_tol_ = 1 is equivalent to the Bayes factor [67], which is used to quantify evidence between two competing models.

**Fig 2:**
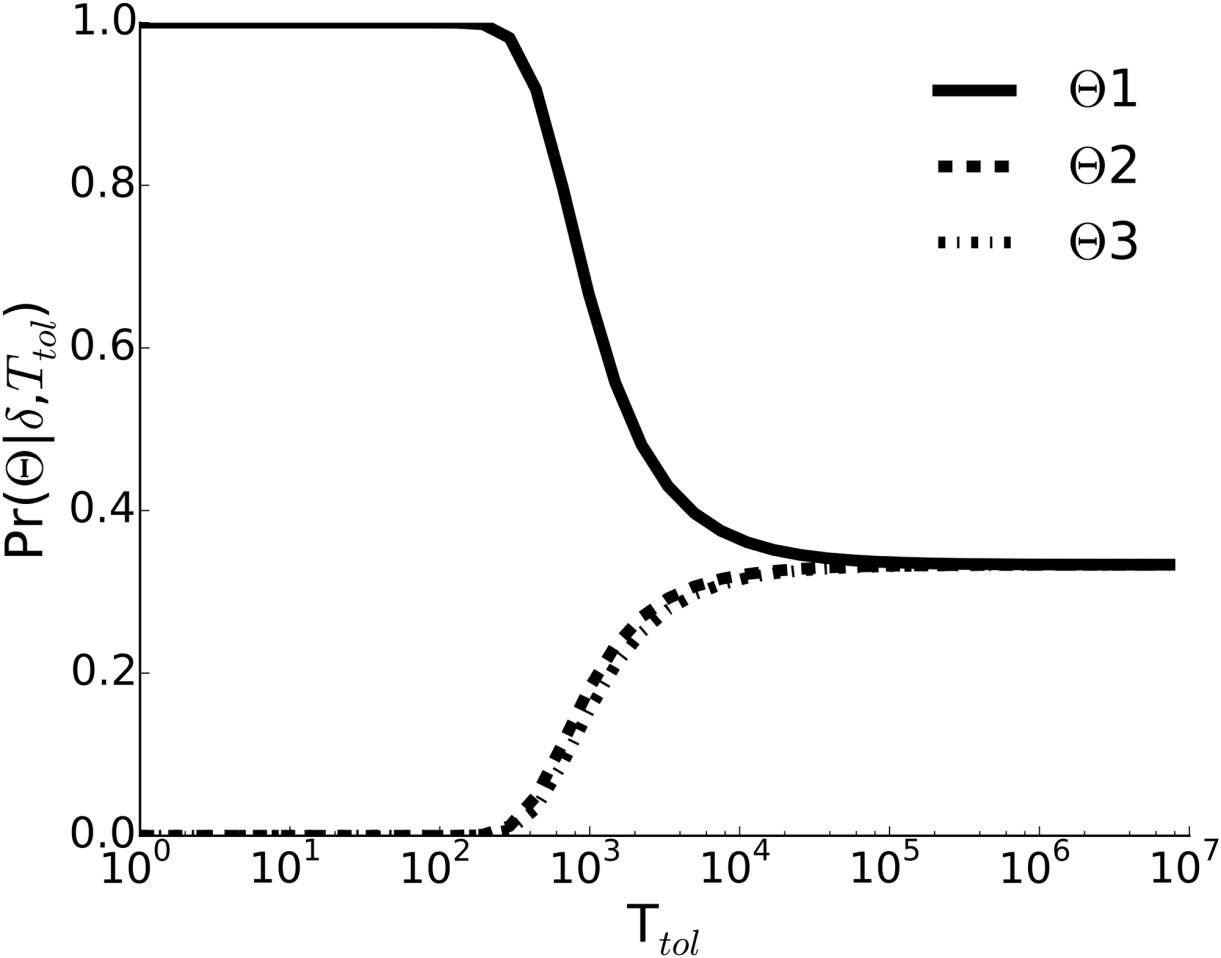
Relative support for the inferred higher-probability posterior modes. Expected posterior probabilities of the selected modes, S={Θ1…Θ3}, are shown against a range of tolerance values, *T*_tol_.

Using the hoppMCMC algorithm [65], we identified a set of posterior modes, S={Θ1…Θ3}, closely matching the Emilia-Romagna surveillance data under the restrictions imposed by data collected from the literature. We assessed the relative support (Eq 7) for each of these modes for a range of *T*_tol_. In Fig 2, we see that Θ1 performs consistently better than the others. Although high *T*_tol_ results in a decline in discriminating ability, there is strong evidence for Θ1 at low tolerances.

In order to quantify the correlation between the simulations and the surveillance data, we calculated expected model output,

$$\begin{array}{c} \bar{y}\left(r,\omega ,\Theta \right)=\frac{1}{N_{\Theta }}\sum_{i=1}^{N_{\Theta }}y\left(r,\omega ,\Theta_{i}\right), \end{array}$$ (8)

where *y*(*r*, *ω*, Θ_*i*_) is a simulation performed with the *i*^*th*^ parameter vector of Θ (see Methods). We obtained a significant association between data and simulations in each province with Θ1 and Θ2, and the overall association was highly significant with all posterior modes (Table 1). In addition, the model slightly underestimated egg counts with any of the posterior modes. On average, we obtained a residual of 69.5, 67.4 and 78.9 with Θ1, Θ2 and Θ3, respectively.

**Table 1:** Agreement between the observation and the simulations. Pearson correlation coefficients (*ρ*) are presented with p-values adjusted with Benjamini & Hochberg multiple test correction.

Province	Θ1	Θ2	Θ3
**Bologna**	0.881*	0.883*	0.846*
**Ferrara**	0.629*	0.659*	0.583*
**Modena**	0.786*	0.795*	0.719*
**Piacenza**	0.746*	0.314^+^	0.144
**Parma**	0.607*	0.539*	0.346^+^
**Ravenna**	0.505*	0.499*	0.562*
**Reggio Emilia**	0.744*	0.498*	0.399^+^
**All data points**	0.690*	0.627*	0.582*

^+^: *p* < 0.05,

*: *p* < 0.001.

**Fig 3:**
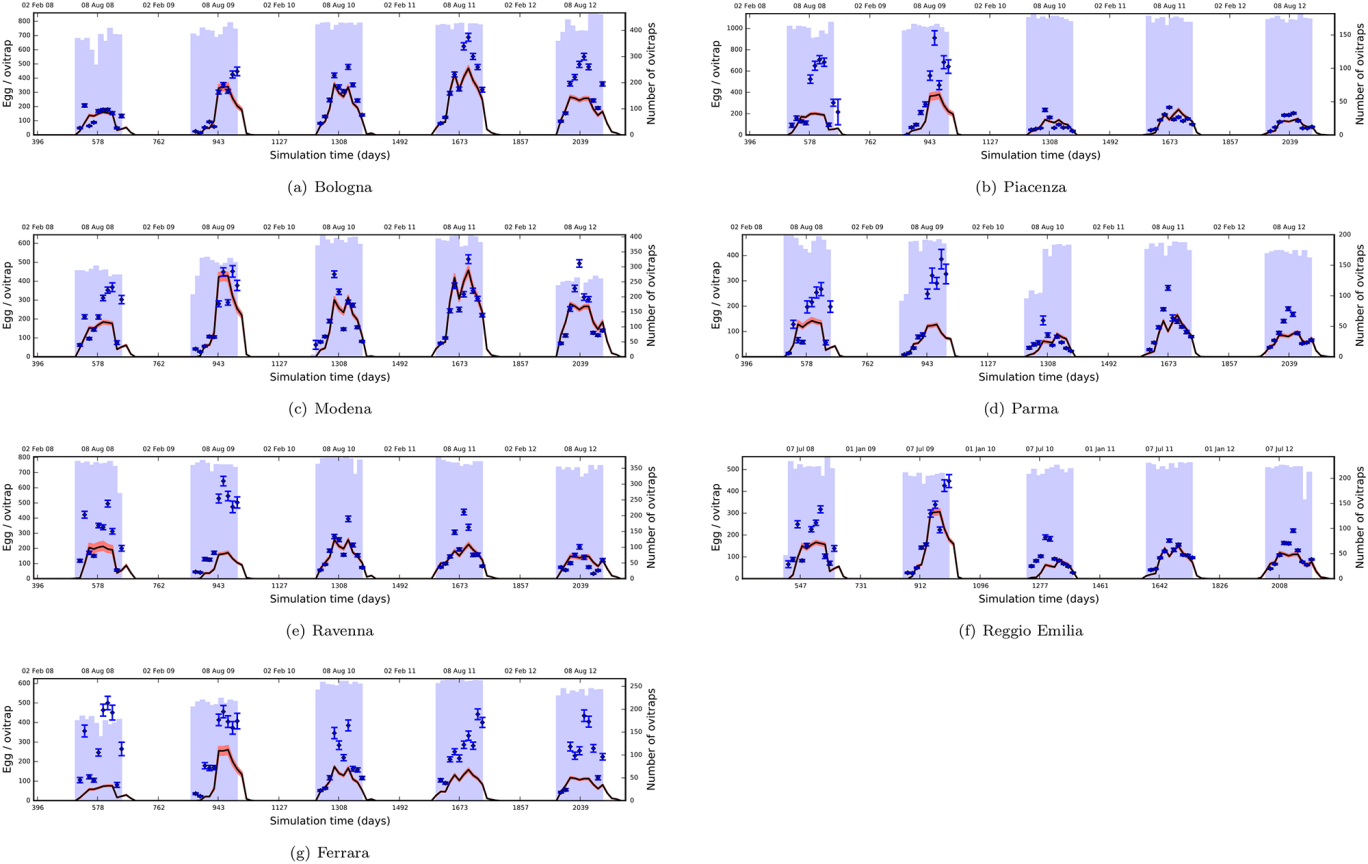
Performance evaluation against the surveillance data from Emilia-Romagna. Blue diamonds represent average egg counts per ovitrap, and vertical error bars represent the standard error of the mean. They are positioned at the dates of data collection along the horizontal axis. Blue bars in the background indicate the number of ovitraps covering a period of two weeks prior to each collection. Solid black lines show model output using the parameters from Θ1 (see text). Red shades represent the 95% confidence interval.

It is important to note that we made no additional effort to distinguish between the provinces or the mosquito high-seasons other than utilising the four types of environmental variables: photoperiod, daily average temperature, daily precipitation and human population density. In Fig 3, we present a comparison of the surveillance data and the simulations with Θ1 (see S4 Fig for the other modes). The figure demonstrates the strong predictive ability of the model through five mosquito high-seasons and for all provinces despite being simulated with the same initial conditions and the same parameters.

The figure shows an apparent separation between the first two and the last three years of the surveillance data. This is especially evident in Piacenza, Parma and Reggio Emilia in Figs 3b, 3d and 3f, respectively, where the egg counts were higher at first, but lower in the final three seasons. This could be a discrepancy due to some environmental dependency which we have yet to capture and incorporate into the model. Alternatively, this could be due to some hidden factors interfering with the data quality, which are currently under investigation [68].

### Sensitivity to parameters and environmental conditions

By investigating the variability and common features within and between posterior modes, it is possible to identify the adaptations required to simulate *Ae. albopictus* population dynamics on a large scale with gridded environmental variables. Differences between prior and posterior probabilities could also serve to validate model assumptions and provide useful information for model improvement.

Firstly, we analysed parameter sensitivity as described in Gutenkunst (2007) [69] and Erguler and Stumpf (2011) [70]. Given $\hat{\theta }$ as the parameter maximising a posterior mode, we approximated the posterior with a multivariate Gaussian around $\hat{\theta }$. The sensitivity matrix, H, can then be estimated using the inverse of variation normalised by $\hat{\theta }$,

$$\begin{array}{c} H=\text{diag}{\left(\hat{\theta }\right)}^{T}\;\Sigma^{-1}\;\text{diag}\left(\hat{\theta }\right), \end{array}$$ (9)

where diag indicates a diagonal matrix with diagonal elements as $\hat{\theta }$. The sensitivity matrix is an indicator of how much and along which dimension we can perturb a parameter vector without significantly affecting posterior probability. Sensitivity for a particular parameter dimension, $H_{\theta_{i}}$, for instance for CPP or *T*_crt_, is defined as the *i*^*th*^ diagonal element of H, *i.e*. $H_{ii}$.

**Fig 4:**
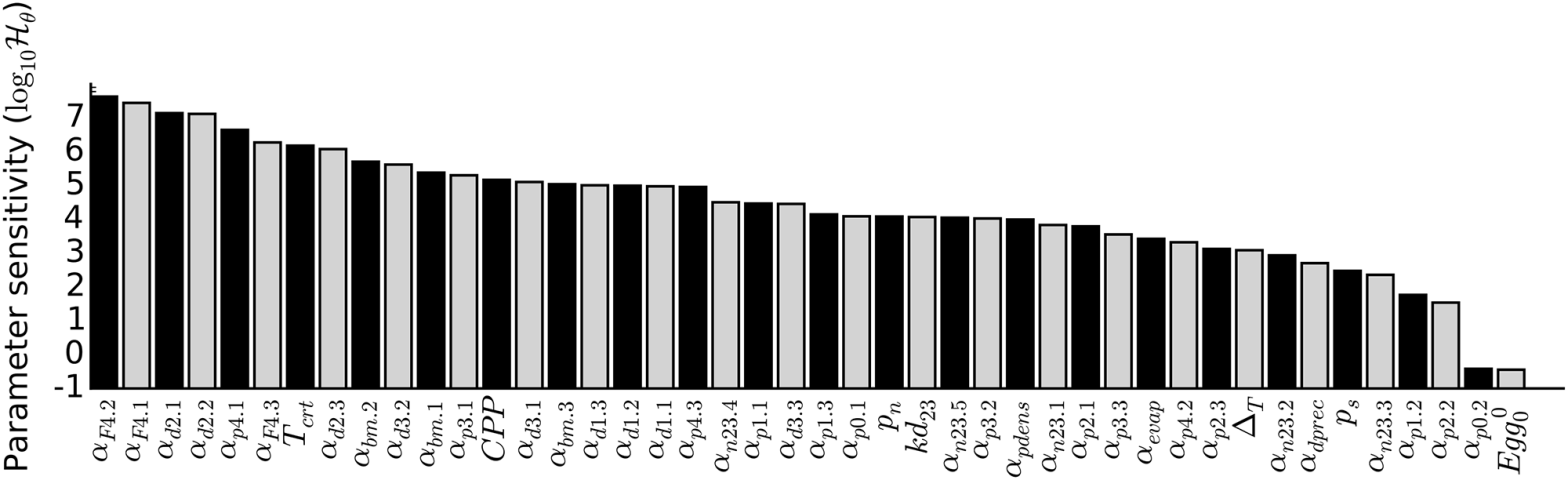
Sensitivity analysis for Θ1. The bar chart displays the logarithm of sensitivity, $H_{\theta }$, along the set of parameter dimensions included in the inference.

In Fig 4, we present parameter sensitivities estimated for the posterior mode Θ1 (see S5 Fig for the other modes). We found that model performance is sensitive predominantly to the set of parameters controlling fecundity. Sensitivity to the parameters controlling development time, including time to first blood meal, is higher than most of the parameters associated with survival. The dynamics is also highly sensitive to *T*_crt_ and CPP. On the other hand, the dynamics is relatively robust to the initial number of diapausing eggs and to *α*_*p*0.2_, which dictates the extend of cold resistance in diapausing eggs. We also note that parameters controlling carrying capacity tend to have relatively low sensitivities.

Parameter sensitivity analysis is indicative of the degree of support each parameter—and associated process—receives with regards to the prior and surveillance data. In addition, we examined the differences between prior and posterior distributions. Since the posterior distribution is informed by field observations, any deviation from the prior demonstrates the permissive values and restrictions required for matching the observations.

**Fig 5:**
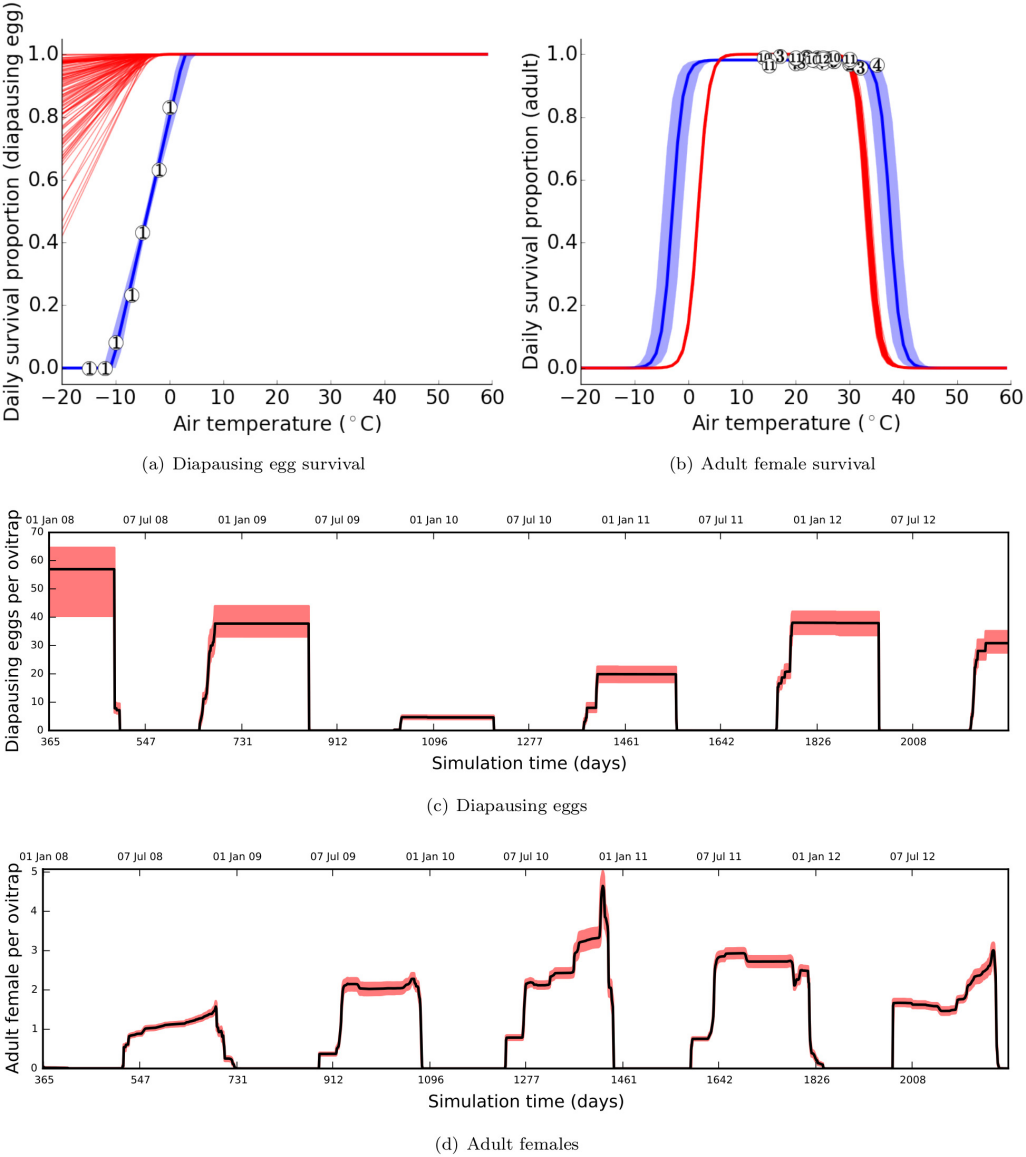
Cold resistance for adults and diapausing eggs with Θ1. Prior (blue) and posterior (red) distributions of diapausing eggs (a) and adult females (b) are shown with data from literature (numbered circles). The prior distribution is represented by a solid line (mean) and a shaded region (95% confidence interval). The posterior distribution is represented by 100 samples drawn from the posterior mode Θ1. Daily average diapausing eggs (c) and adult females (d) per ovitrap are simulated with Θ1. The solid line represents the mean and the red shade represents the 95% confidence interval. (a) 1: [52], (b) 1: [71], 2: [72], 3: [58], 4: [46], 5: [73], 6: [74], 7: [75], 8: [76], 9: [77], 10: [78], 11: [79], 12: [80].

One of the main differences for Θ1 is the extent of cold resistance in diapausing eggs. In this posterior mode, in order to explain the survival of *Ae. albopictus* populations through harsh winter conditions, the model necessitates that diapausing eggs be more resistant to negative temperatures than observed in the laboratory. Although such temperatures are daily averages represented by environmental grid points, such a requirement implies that diapausing eggs survive through winter almost unharmed (see Fig 5a and 5c). On the other hand, adults, which are not as cold resistant as diapausing eggs, do not survive through winter (see Fig 5b and 5d).

The case for Θ2 and Θ3 is the opposite, for which cold resistance is required not for diapausing eggs but for adults (see S6 Fig). With these modes, the model predicts that cold resistant adults help to carry *Ae. albopictus* populations from one mosquito season to another. While they survive through winter, they are in a low-activity state: they do not take blood meals or lay eggs (see S7 Fig). In this case, populations continue laying diapausing eggs, however, a large fraction is lost through winter (see S8 Fig). However, since Θ2 and Θ3 have lower probabilities (Fig 3) and weaker association with data (Table 1) compared to Θ1, it is less likely to have cold resistant adults for winter survival.

Next, we investigated the different mechanisms for the regulation of diapause proposed by each posterior mode. The thresholds of photoperiod (CPP) and temperature (*T*_crt_) for entry into and exit from diapause can be seen in Table 2. These thresholds, when applied to the gridded environmental datasets, yield diapausing from early October to early March with Θ1 and Θ2. For Θ3, the period is longer, from late August to late April. According to the reports of Lacour and coworkers, egg diapause in Nice takes place from late September to early March [43, 81], which agrees well with the predictions of Θ1 and Θ2.

**Table 2:** Photoperiod (CPP) and temperature (*T*_crt_) thresholds for each posterior mode.

Thresholds	Θ1	Θ2	Θ3
**Photoperiod (hours)**	11.60 ± 0.05	11.20 ± 0.02	13.56 ± 0.07
**Temperature (°*C*)**	17.40 ± 0.02	28.29 ± 0.01	24.92 ± 0.02

As described in the Methods, the duration of simulation extends over six years on 23 grid points, which covers the area of surveillance in the seven provinces of Emilia-Romagna. A closer look at the initiation and termination of diapause during this period reveals that exit from diapause is determined by an increase in day length regardless of which posterior mode is chosen (see Table 3). In all cases, at the times when photoperiod exceeds the CPP, *T*_crt_ has already been exceded. On the other hand, diapause initiation is mostly controlled by a decline in air temperature rather than photoperiod for Θ1. In more than 75% of the cases, day length is already shorter than the CPP when temperature drops below *T*_crt_. For Θ2, initiation is controlled only by photoperiod as in the case of termination. For Θ3, initiation is controlled either by photoperiod or temperature in about 40% of the cases. In about 15–20% of the cases, for Θ1 and Θ3, both photoperiod and temperature exceed their respective thresholds at the same time triggering diapause initiation.

**Table 3:** Environmental dependency of diapause control as predicted by Θ1, Θ2 and Θ3. The numbers are percentages over all entry or exit events encountered during the six years of simulation.

	Θ1		Θ2		Θ3	
	Entry	Exit	Entry	Exit	Entry	Exit
**Photoperiod**	11.98	100	100	100	38.33	100
**Temperature**	75.77	0	0	0	43.58	0
**Both**	12.25	0	0	0	18.09	0

One of the common characteristics of the three posterior modes is high sensitivity to the development times of the immature stages (S6 Fig). Despite high sensitivity, largest deviations from prior expectation are observed in development especially for Θ1. Overall, development times for immature stages are higher than expected and they tend to increase with temperature.

Although this may indicate a discrepancy between laboratory and field populations, one must be cautious with interpreting such differences in strict biological context. Since a grid point represents an average over the region it covers, a certain degree of association can be expected between this average and the breeding sites in the region represented by the point. However, direct biological interpretation would be meaningful only if there is a strong correspondence. This is unlikely at large scales with various landscapes and microhabitats.

We argue that the average daily temperature data is also a substantial contributor to the observed differences. It is well-known that the activity of adult *Ae. albopictus* peaks at dusk and dawn [20], which suggests that different life stages might be affected by environmental conditions at different times during the diel cycle. Although this is not captured when dealing with daily averages, the deviations from the prior seen in the posterior modes indicate the adaptations required to match the observations using the available environmental datasets.

In addition to temperature, rainfall is expected to have an effect on vector populations causing the expansion of possible breeding sites [15]. Θ1 predicts a highly volatile carrying capacity meaning that the inferred value of *α*_*evap*_ is too low to maintain K for long periods—breeding sites lose most of their capacity in a few days. Since the surveillance mainly focused on urbanised areas [64], Θ1 suggests that carrying capacity is highly dependent on short-term rainfall activities even in urbanised areas (see S9 Fig). On the other hand, Θ2 and Θ3 predict a higher *α*_*evap*_, lower volatility, allowing longer-term retention of carrying capacity. This renders the capacity less dependent on sort-term variations in rainfall; however, higher *α*_*evap*_ results in higher variability in K.

Alternative models of breeding sites have been widely used [30, 43, 49, 50]; however, to the best of our knowledge, none of the previous studies considered the possible effects of both human populations and rainfall in their models. Also, none of the models, including the one presented here, can be calibrated against relevant observations since detailed information on breeding sites is not recorded in surveillance studies. Since there is a strong dependency of population dynamics on carrying capacity, more data are needed on breeding sites and their dependency on human activities and precipitation to develop more accurate models.

We note here that the region of surveillance offers only a limited range of environmental conditions. This has enabled us to constrain environmental dependency to a moderate degree. In order to improve the model and its environmental dependency, in addition to the laboratory data, field observations on populations adapted to different climates and different levels of urbanisation are required.

### Evaluating validity over Europe

In previous sections, we described the development of an environmentally-driven population dynamics model of *Ae. albopictus* using gridded environmental datasets. We demonstrated its applicability over the region of inference, Emilia-Romagna, Italy. Eventually, we aim to be able to predict environmental suitability for *Ae. albopictus* populations outside this region and anywhere over the globe. Prompted by the success of the three posterior modes, especially of Θ1, here we test model validity over Europe.

Instead of seeking an absolute measure of habitat suitability, we compared relative sizes of vector populations between Emilia-Romagna and a point of interest. Employing the same assumptions, the same parameter set—including the initial conditions—we simulated the daily average number of adult females from 2007 until the end of 2012. For instance, using the model with Θ1, we predicted that on average there was about 1 adult female *Ae. albopictus* per ovitrap per day in Bologna. For Parma, the predicted average was about half of this (0.437 adult females per ovitrap per day).

We defined the reference suitability index (RSI) as the minimum of the set of daily average adult female counts within the surveillance region. Evidently, even at the province with the minimum vector population size, the environmental conditions were suitable to promote vector establishment. We argue that comparing predicted population sizes with this index will inform about the relative population size and the suitability of environmental conditions under the same assumptions as for the surveillance region.

We found that, the RSI predicted with Θ1 was 0.437, while for Θ2 and Θ3, it was 0.811 and 0.456, respectively. With Θ1 and Θ3, the minimum population size was predicted for Parma, and with Θ2 for Piacenza. We found that the model predicts higher vector counts overall when simulated with Θ2.

Based on the RSI, we defined the habitat suitability index (HSI) as the ratio of a predicted population size to RSI. If the HSI is larger than 1 for a given grid point, a higher population size and, therefore, a higher environmental suitability should be expected for the region. If the HSI is lower than 1, this indicates a lower environmental suitability; however, the region may still be able to support the establishment of *Ae. albopictus* populations. For practical reasons, in this context, we defined an arbitrary HSI threshold of 1/16 below which we consider a region unsuitable for *Ae. albopictus* populations.

**Fig 6:**
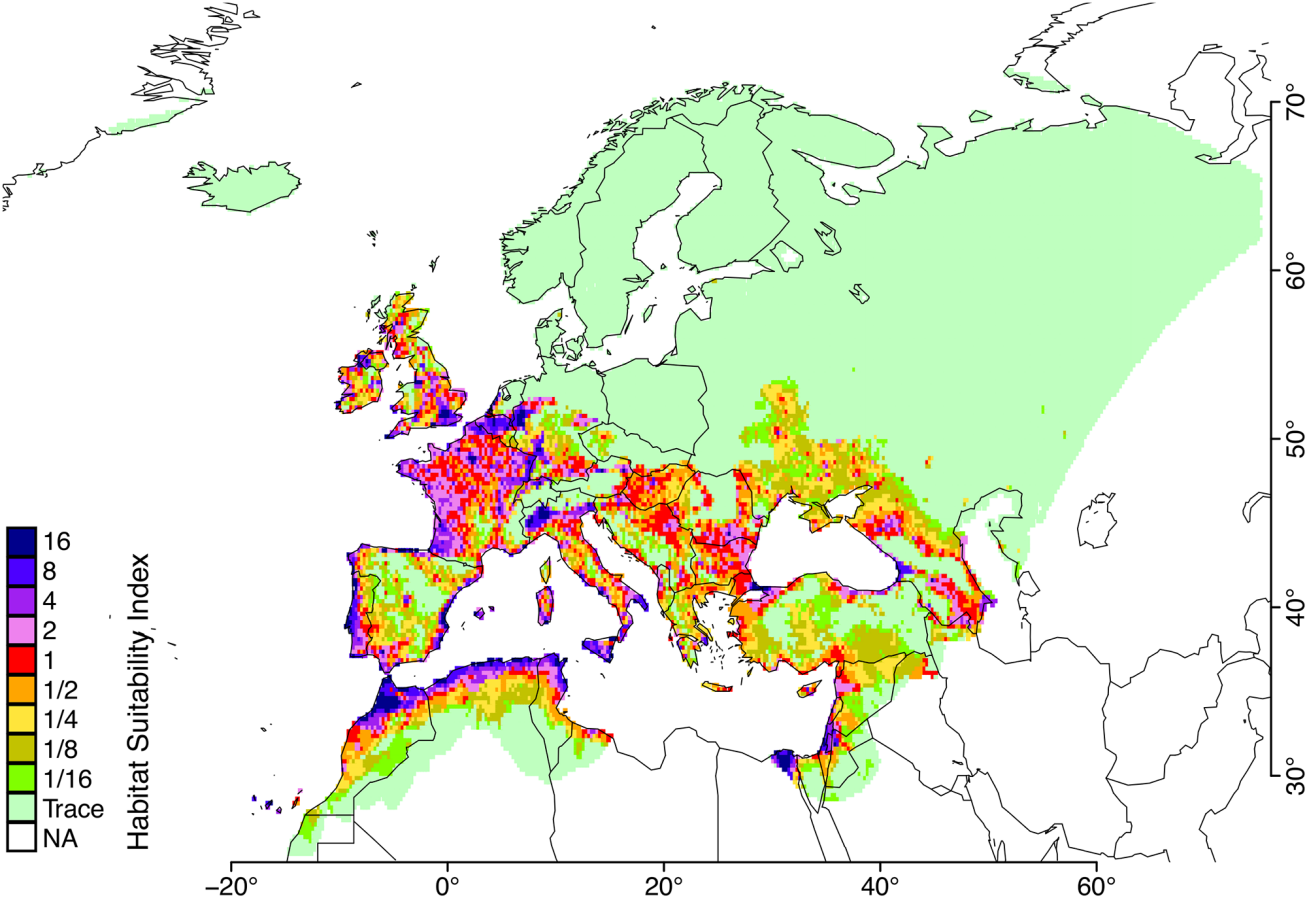
Habitat suitability indices (HSI) for Europe with Θ1. The indices are shown for the available grid points of the environmental datasets as described in the text. Each value indicates the expected fold change compared to the reference index (RSI) obtained over Emilia-Romagna.

In Fig 6, we present the HSI derived with Θ1 for all the grid points in the environmental datasets (sec. Environmental data). Habitat suitability derived with the other posterior modes can be seen in S10 Fig. Overall, the model with Θ1 gives emphasis to the suitability of the Mediterranean coastline, and assigns high suitabilities for France, Belgium and the UK as the northernmost territories. We observed that suitability predicted with Θ1 was similar to the predictions reported in Benedict *et al*. 2007 [82], Fisher *et al*. 2011 [83] and Proestos *et al*. 2015 [28].

Various methodologies were involved in many studies aiming for predicting environmental suitability for *Ae. albopictus* populations. An elaborate comparison with previous predictions is the subject of future research; however, despite being qualitative, the similarity observed is reassuring in the sense that the model with Θ1 adheres to the statistical inferences previously drawn from large-scale studies.

In order to evaluate predictions with different posterior modes, we employed the most recent surveillance reports of the VBORNET [84]. The data from the July 2015 release were available to us at the NUTS3 resolution. To enable direct comparison we extracted geolocations of the European NUTS3 territories from the GISCO NUTS 2010 dataset (GISCO-Eurostat, European Commission), and grouped environmental grid points for each geolocation. We assumed that the largest HSI is the most representative of each group.

We note that *Ae. albopictus* has been constantly expanding its range in Europe to date [84]. While the reports represent a snapshot in time, the spread of the vector is a dynamic process leaving a wide window of uncertainty over the territories where it is currently absent. It is likely that certain territories, where the vector is currently absent, might be suitable for establishment, and will host *Ae. albopictus* populations in the near future. Therefore, we focused explicitly on the regions with already established vector populations, and asserted that the model predicts high suitability for a large fraction of these regions.

As seen in Table 4, the model with Θ1 successfully assigns about 90% (247 out of 272) of all vector-established territories a high suitability index, *i.e*. HSI≥1. Only 1 of these territories was deemed unsuitable (labelled as trace). This is a small mountainous province, named Sondrio (ITC44), located at the northern border of Italy. We argue that the environmental datasets might not be at a sufficient resolution suitable for this region. On the other hand, both the habitat suitability plots (Fig 6) and evaluation with the VBORNET reports (Table 4), indicate that Θ2 and Θ3 do not capture the presence of the vector in Europe as well as Θ1 does.

**Table 4:** Validity of the habitat suitability analysis with respect to the surveillance reports of VBORNET.

%	Trace	1/16	1/8	1/4	1/2	≥ 1
Θ1	0.4	0	0.7	5.1	2.9	90.8
Θ2	12.5	0	1.1	0.4	4	82
Θ3	4.8	3.7	3.7	2.2	11.4	74.3

Since the model is calibrated with the data from Italy, the habitat suitability maps show possible range expansion specifically for the Italian *Ae. albopictus* population. In order to improve the accuracy of environmental dependence, surveillance data from different habitats with varying climates and degrees of urbanisation should be combined. Data from stable vector populations are ideal for this purpose. For instance, we expect the vector distribution in Asia to have reached climatic limits and be adapted to local climate conditions. With such data, environmental conditions limiting vector establishment can be defined clearly without the interference of range expansion. Unfortunately, we do not have presence or absence data at the present time from such stable populations; however, we aim to perform this analysis when such data become available.

## Conclusion

Major health concerns have emerged in recent years as *Ae. albopictus* populations are rapidly expanding their range towards temperate climates in Europe and America. Understanding the environmental dependence of the vector is key to the understanding of the limits of this expansion. In addition, availability of a valid population dynamics model enables more accurate projections of future *Ae. albopictus* populations, and also helps to identify voids in our understanding of key biological processes.

We studied the environmental dependence of *Ae. albopictus* population dynamics by developing a mathematical model. The model incorporates temperature, precipitation, human population density and photoperiod as the main environmental drivers. We performed Bayesian inference on all model parameters accounting for laboratory observations from the literature and vector surveillance data from Emilia-Romagna, Italy. With the inference, we ensured optimum adherence to empirical biological constraints while linking the dynamics to globally available gridded environmental datasets.

As a result, we found that the model demonstrates high predictive skill over the region of inference and beyond, confirming most of the current reports of vector presence in Europe. We identified a set of best and alternative explanations in terms of different parameter configurations, and examined various aspects of environmental dependence. We examined the mechanism of diapause and inferred that exit from and entry into diapause are differentially controlled with temperature and photoperiod. Although not having incorporated explicit instructions for the dates of diapause initiation and termination, the inference arrived at close approximations to the observations from the literature. The model indicates that diapausing eggs are considered more resistant to negative temperatures when using gridded environmental variables compared to laboratory conditions. Under such conditions, only diapausing eggs are predicted to survive through winter and carry the vector population to the next suitable season.

Despite the remarkable performance of the model, the possibility of a better posterior mode cannot be discarded completely. In order to perform better inferences and to capture various aspects of environmental dependence, the observational datasets must be expanded. Most importantly, the surveillance period may be extended a few weeks before and after the mosquito high-season, and a combination of ovitrap and adult mosquito traps may be employed in surveillance. Annual profiles of adult vectors might help to discriminate between alternative explanations and to draw better inferences on the environmental dependence of diapause. In addition to observing different life stages, surveillance data from a broad range of *Ae. albopictus* populations with known adaptation to different levels of urbanisation and dependence to rainfall and photoperiod would help improve the accuracy and applicability of the model. We believe that the Bayesian approach and the procedure of model construction introduced in this manuscript are highly advantageous and immediately applicable given the availability of such data.

## Supporting Information

S1 File The albopictus package for Python (v2.7) implementing the model and the user interface. (GZ)

S1 Text Derivation of the scaled carrying capacity. (PDF)

S2 Text Instructions for installing and using the albopictus package. (TXT)

S1 Table Model parameters with functional forms of environmental dependence and prior distributions. Median, blue line, and 95% confidence range, blue shade, of prior distributions are also plotted to aid in visualisation.
(PDF)

S1 Fig Temperature- and density-dependent development of the immature stages. The blue surface indicates the expected duration of larva and pupa, (*d*_2_+*d*_3_)*d*_23_, with respect to water temperature, *T*_*w*_, and density of the immature stages, *μ*. Observed duration of the immature stages are given as white circles and the difference between observed and expected values are marked with horizontal red lines. 1: Gavotte *et al*. 2009, 3: Delatte *et al*. 2009, 4: Liu 1965, 5: Halcrow 1955, 6: Udaka 1959 (see S1 Table for the references).
(PDF)

S2 FigDecision making for the diapausing behaviour.The diagram outlines the algorithm with which the indicator functions, $I_{\text{fav}}$ and $I_{\text{unfav}}$, determine suitable conditions for diapausing and egg hatching with respect to temperature and photoperiod.(PDF)

S3 Fig Grid points of the higher-resolution datasets used for parameter inference. (PDF)

S4 Fig Evaluating model performance over Emilia-Romagna with Θ1, Θ2 and Θ3. (PDF)

S5 Fig Sensitivity analysis for Θ1, Θ2 and Θ3. (PDF)

S6 Fig Comparison of prior and posterior distributions Θ1, Θ2 and Θ3. The prior distribution is represented by a solid line (mean) and a shaded region (95% confidence interval). The posterior distribution is represented by 100 samples drawn from each posterior mode. Data from literature are plotted as numbered circles (see S1 Table for the references). Figures are grouped in three columns, one for each posterior mode, Θ1, Θ2 and Θ3.
(PDF)

S7 Fig Simulated number of adult females per ovitrap. Solid black line shows the mean while red shade indicates the 95% confidence interval. Figures are grouped in three columns, one for each posterior mode, Θ1, Θ2 and Θ3.
(PDF)

S8 Fig Simulated number of diapausing eggs per ovitrap. Solid black line shows the mean while red shade indicates the 95% confidence interval. Figures are grouped in three columns, one for each posterior mode, Θ1, Θ2 and Θ3.
(PDF)

S9 FigSimulated scaled carrying capacity, K, per ovitrap.Solid black line shows the mean while red shade indicates the 95% confidence interval. Figures are grouped in three columns, one for each posterior mode, Θ1, Θ2 and Θ3.(PDF)

S10 Fig Habitat suitability indices (HSI) for Europe with Θ1, Θ2 and Θ3. (PDF)
