# Supplementary material for: Large-Scale Modelling of the Environmentally-Driven Population Dynamics of Temperate Aedes albopictus (Skuse)
Source: PLoS One. 2016 Feb 12;11(2):e0149282. doi: 10.1371/journal.pone.0149282 (PMC4752251; doi:10.1371/journal.pone.0149282)
Supplement: S1 Text — (PDF) [file pone.0149282.s002.pdf]

Supporting Text S1: Carrying Capacity

In White *et al.*, 2011, Equation 10, carrying capacity is defined as proportional to the average of previous precipitation events weighted by an exponential distribution with mean τ days [1]. According to this,

$$K(t) = \lambda \frac{1}{\tau \left(1 - e^{-t/\tau}\right)} \int_0^t e^{-(t-t')/\tau} R(t') dt',$$

where $R(t)$ represents rainfall at time t.
When only daily average precipitation is available, it is possible to assume that the amount of precipitation for a day is constant and equal to the daily average. Under this assumption, we transform the equation to discrete-time with daily time-steps,

$$K(t) = \lambda \frac{1}{\tau \left(1 - e^{-t/\tau}\right)} \sum_{x=1}^{t} R(x) \int_{x-1}^{x} e^{-(t-t')/\tau} dt',$$

for $t = 1, 2, \ldots$ days. This is equivalent to

$$K(t) = \lambda \frac{1}{\tau \left(1 - e^{-t/\tau}\right)} \sum_{x=1}^{t} R(x)\, e^{-(t-x)/\tau} \int_{t-1}^{t} e^{-(t-t')/\tau} dt',$$

where,

$$\int_{t-1}^{t} e^{-(t-t')/\tau} dt' = \tau \, (1 - e^{-1/\tau}).$$

As a result,

$$K(t) = \lambda \frac{1}{\tau \, (1 - e^{-t/\tau})} \, \tau \, (1 - e^{-1/\tau}) \sum_{x=1}^{t} e^{-(t-x)/\tau} R(x),$$

and

$$K(t) = \lambda \frac{1 - e^{-1/\tau}}{1 - e^{-t/\tau}} \sum_{x=1}^{t} e^{-(t-x)/\tau} R(x).$$

In addition to rainfall, it is possible to incorporate human activities to this equation as a contribution to carrying capacity. The equation for K can, then, be written as

$$K(t) = \frac{1 - e^{-1/\tau}}{1 - e^{-t/\tau}} \sum_{x=1}^{t} e^{-(t-x)/\tau} \left[\lambda_d p_d(x) + \lambda_R R(x)\right].$$

The sum in this equation can be written as a time-difference equation with which the capacity is calculated by daily iterations. In essence,

$$k(t) = \lambda_d p_d(t) + \lambda_R R(t) + e^{-1/\tau} k(t-1), \quad \text{where} \quad k(0) = 0,$$

and

$$K(t) = \frac{1 - e^{-1/\tau}}{1 - e^{-t/\tau}} \, k(t).$$

If we let $\mathcal{B} = k$, $\alpha_{evap} = e^{-1/\tau}$, $\alpha_{pdens} = \lambda_d$ and $\alpha_{dprec} = \lambda_R$, the equation becomes equivalent to the scaled carrying capacity defined in the main text.

$$\mathcal{B}(t) = \alpha_{pdens} p_d(t) + \alpha_{dprec} R(t) + \alpha_{evap} \mathcal{B}(t-1), \quad \text{where} \quad \mathcal{B}(0) = 0,$$

and

$$\mathcal{K}(t) = \frac{1 - \alpha_{evap}}{1 - \alpha_{evap}^{t}} \, \mathcal{B}(t).$$

References

1. White MT, Griffin JT, Churcher TS, Ferguson NM, Basáñez MG, et al. (2011) Modelling the impact of vector control interventions on anopheles gambiae population dynamics. Parasit Vectors 4: 153.
2. Christiansen-Jucht C, Erguler K, Shek CY, Basáñez MG, Parham PE (2015) Modelling anopheles gambiae s.s. population dynamics with temperature- and age-dependent survival. IJERPH 12: 5975–6005.
