# Supplementary material for: Large-Scale Modelling of the Environmentally-Driven Population Dynamics of Temperate Aedes albopictus (Skuse)
Source: PLoS One. 2016 Feb 12;11(2):e0149282. doi: 10.1371/journal.pone.0149282 (PMC4752251; doi:10.1371/journal.pone.0149282)
Supplement: S1 Table — Median, blue line, and 95% confidence range, blue shade, of prior distributions are also plotted to aid in visualisation. (PDF) [file pone.0149282.s004.pdf]

Table S.1. Model parameters with functional forms of environmental dependence and prior distributions. Median, blue line, and 95% confidence range, blue shade, of prior distributions are also plotted to aid in visualisation.

Model Parameters	Description	Prior Distribution	References
Egg_0	Initial number of non-diapausing eggs per ovitrap	0 (fixed)	
Egg_0^*	Initial number of tagged eggs per ovitrap	0 (fixed)	
Egg_0^0	Initial number of diapausing eggs per ovitrap	$\text{Uniform}(0, 10^6)$	
Larva_0	Initial number of larvae per ovitrap	0 (fixed)	
Pupa_0	Initial number of pupae per ovitrap	0 (fixed)	
Naive_0	Initial number of naive females per ovitrap	0 (fixed)	
Adult_0	Initial number of adult females per ovitrap	0 (fixed)	
α_{pdens}	Increase in carrying capacity per unit human population density per ovitrap per day	$\text{Uniform}(0, 1)$	
α_{dprec}	Increase in carrying capacity per unit precipitation per ovitrap per day	$\text{Uniform}(0, 1)$	
α_{evap}	Fraction of carrying capacity retained daily	$\text{Uniform}(0, 1)$	
CPP	Critical photoperiod (photoperiod: fraction of the day with daylight)	$\text{Uniform}(0, 1)$	
T_{crt}	Critical air temperature for laying diapausing eggs	$\mathcal{N}(21, 3)$	[1,2]
p_s	Fraction of eggs tagged for diapause each day	$\text{Uniform}(0, 1)$	
p_n	Fraction of diapausing eggs exiting diapause each day	$\text{Uniform}(0, 1)$	
Δ_T	$T_w - T_a$: The difference between water and air temperature	$\mathcal{N}(0, 3)$	

Table S.1 – continued from previous page

Model Parameters	Description	Prior Distribution	References
$\alpha_{d1.1}$<br>$\alpha_{d1.2}$<br>$\alpha_{d1.3}$	Average development time for eggs<br>**Functional form:**<br>$d_1(T_w) = \alpha_{d1.1} + \alpha_{d1.2}\, T_w + \alpha_{d1.3}\, T_w^2$<br>**Range:** $[0, \infty)$	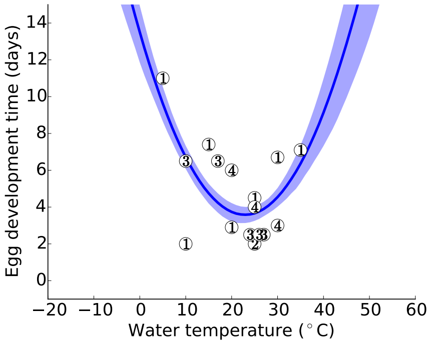	1: [4]<br>2: [41]<br>3: [27]<br>4: [28]
$\alpha_{d2.1}$<br>$\alpha_{d2.2}$<br>$\alpha_{d2.3}$	Average development time for larvae<br>**Functional form:**<br>$d_2(T_w) = \alpha_{d2.1} + \alpha_{d2.2}\, T_w + \alpha_{d2.3}\, T_w^2$<br>**Range:** $[0, \infty)$	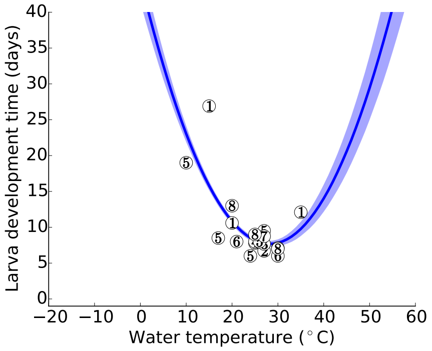	1: [4]<br>2: [18]<br>3: [16]<br>4: [42]<br>5: [19]<br>6: [41]<br>7: [27]<br>8: [43]<br>9: [44]<br>10: [45]
$\alpha_{d3.1}$<br>$\alpha_{d3.2}$<br>$\alpha_{d3.3}$	Average development time for pupae<br>**Functional form:**<br>$d_3(T_w) = \alpha_{d3.1} + \alpha_{d3.2}\, T_w + \alpha_{d3.3}\, T_w^2$<br>**Range:** $[0, \infty)$	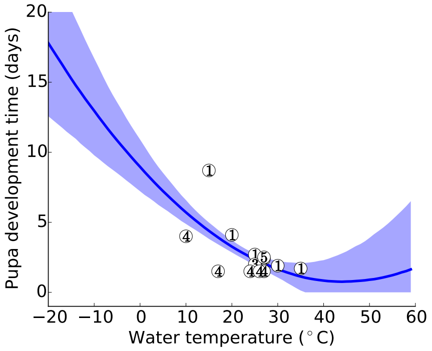	1: [4]<br>2: [42]<br>3: [41]<br>4: [27]<br>5: [44]

Table S.1 – continued from previous page

Model Parameters	Description	Prior Distribution	References
$\alpha_{bm.1}$<br>$\alpha_{bm.2}$<br>$\alpha_{bm.3}$	Time from becoming an adult to obtaining first blood-meal<br>**Functional form:**<br>$t_{bm}(T_a) = \alpha_{bm.1} + \alpha_{bm.2}\, T_a + \alpha_{bm.3}\, T_a^2$<br>**Range:** $[0, \infty)$	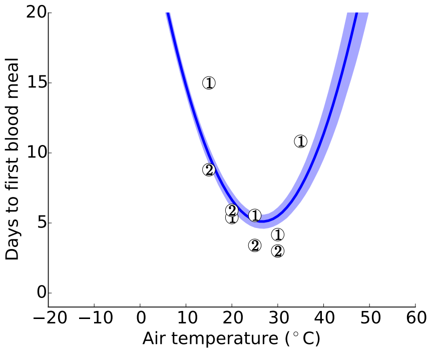	1: [4]<br>2: [6]
$\alpha_{F4.1}$<br>$\alpha_{F4.2}$<br>$\alpha_{F4.3}$	Number of eggs laid per female adult mosquito per day<br>**Functional form:**<br>$F_4(T_a) = \alpha_{F4.1} + \alpha_{F4.2}\, T_a + \alpha_{F4.3}\, T_a^2$<br>**Range:** $[0, \infty)$	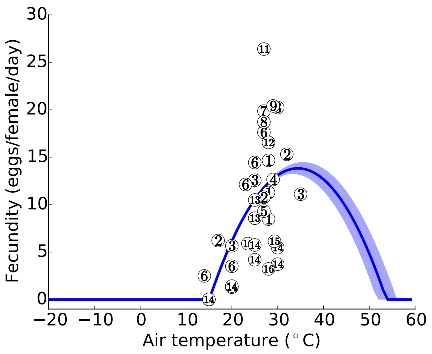	1: [11]<br>2: [22]<br>3: [4]<br>4: [29]<br>5: [26]<br>6: [27]<br>7: [30]<br>8: [31]<br>9: [32]<br>10: [12]<br>11: [33]<br>12: [24]<br>13: [34]<br>14: [23]<br>15: [6]<br>16: [10]

Table S.1 – continued from previous page

Model Parameters	Description	Prior Distribution	References
$\alpha_{p0.1}$<br>$\alpha_{p0.2}$	Daily survival probability of diapausing eggs<br>**Functional form:**<br>$p_0(T_a) = \alpha_{p0.1} + \alpha_{p0.2}\, T_a$<br>**Range:** $[0, 1]$	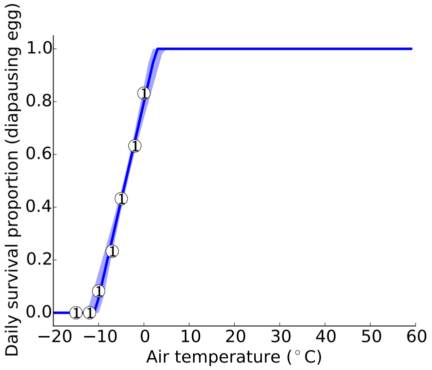	1: [3]
$\alpha_{p1.1}$<br>$\alpha_{p1.2}$<br>$\alpha_{p1.3}$	Daily survival probability of non-diapausing eggs<br>**Functional form:**<br>$p_1(T_w) = \alpha_{p1.1} \frac{1}{1+e^{(\alpha_{p1.2}-Tw)}} \frac{1}{1+e^{(Tw-\alpha_{p1.3})}}$<br>**Range:** $[0, 1]$	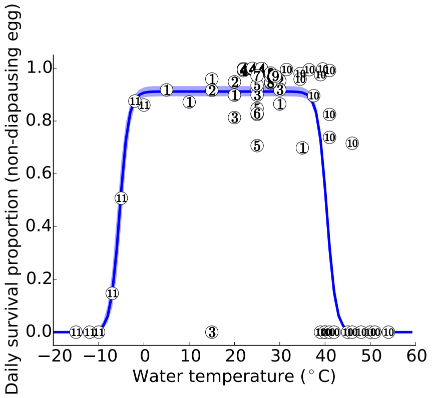	1: [4]<br>2: [5]<br>3: [6]<br>4: [7]<br>5: [8]<br>6: [9]<br>7: [10]<br>8: [11]<br>9: [12]<br>10: [13]<br>11: [3]

Table S.1 – continued from previous page

Model Parameters	Description	Prior Distribution	References
$\alpha_{p2.1}$<br>$\alpha_{p2.2}$<br>$\alpha_{p2.3}$	Daily survival probability of larvae<br>**Functional form:**<br>$p_2(T_w) = \alpha_{p2.1}\frac{1}{1+e^{(\alpha_{p2.2}-Tw)}}\frac{1}{1+e^{(Tw-\alpha_{p2.3})}}$<br>**Range:** $[0, 1]$	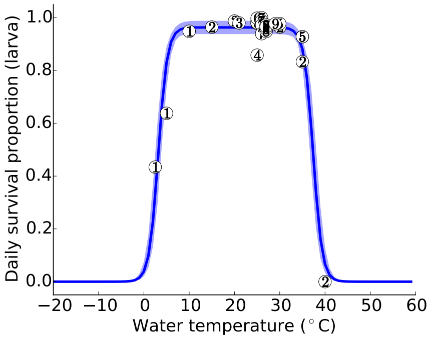	1: [14]<br>2: [4]<br>3: [15]<br>4: [16]<br>5: [17]<br>6: [10]<br>7: [18]<br>8: [19]<br>9: [12]
$\alpha_{p3.1}$<br>$\alpha_{p3.2}$<br>$\alpha_{p3.3}$	Daily survival probability of pupae<br>**Functional form:**<br>$p_3(T_w) = \alpha_{p3.1}\frac{1}{1+e^{(\alpha_{p3.2}-Tw)}}\frac{1}{1+e^{(Tw-\alpha_{p3.3})}}$<br>**Range:** $[0, 1]$	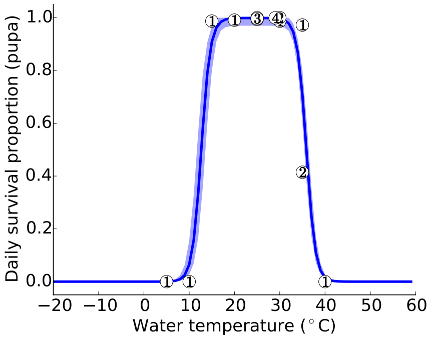	1: [4]<br>2: [17]<br>3: [10]<br>4: [12]
$\alpha_{p4.1}$<br>$\alpha_{p4.2}$<br>$\alpha_{p4.3}$	Daily survival probability of adults<br>**Functional form:**<br>$p_4(T_a) = \alpha_{p4.1}\frac{1}{1+e^{(\alpha_{p4.2}-Ta)}}\frac{1}{1+e^{(Ta-\alpha_{p4.3})}}$<br>**Range:** $[0, 1]$	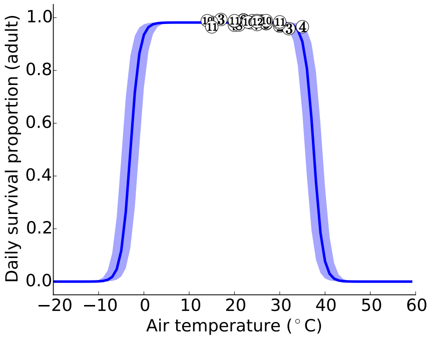	1: [20]<br>2: [21]<br>3: [22]<br>4: [4]<br>5: [23]<br>6: [24]<br>7: [25]<br>8: [15]<br>9: [26]<br>10: [27]<br>11: [6]<br>12: [28]

Model Parameters	Description	Prior Distribution	References
kd_{23}	Density-dependent reduction in the daily survival probabilities of larvae and pupae<br>**Functional form:**<br>$\mu_t = (\text{Larva}_t + \text{Pupa}_t)/\mathcal{K}_t$<br>$p_{LD}(\mu_t) = e^{-kd_{23}\,\mu_t}$<br>**Range:** $[0, 1]$	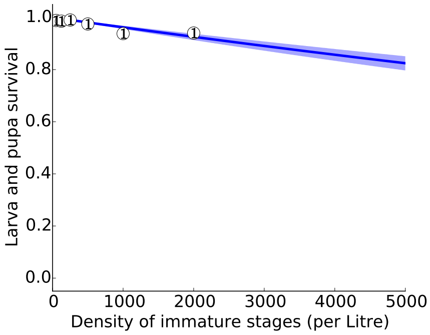	1: [40]
$\alpha_{n23.1}$<br>$\alpha_{n23.2}$<br>$\alpha_{n23.3}$<br>$\alpha_{n23.4}$<br>$\alpha_{n23.5}$	Density- and temperature-dependent increase in development times of larvae and pupae<br>**Functional form:**<br>$\mu_t = (\text{Larva}_t + \text{Pupa}_t)/\mathcal{K}_t$<br>$\tau(T_w) = \alpha_{n23.3} + \alpha_{n23.4}\,T_w + \alpha_{n23.5}\,T_w^2$<br>$d_{23}(\mu_t, T_w) = \alpha_{n23.1}\,\mu_t^{\alpha_{n23.2}\,\tau(T_w)}$<br>**Range:** $[1, \infty]$	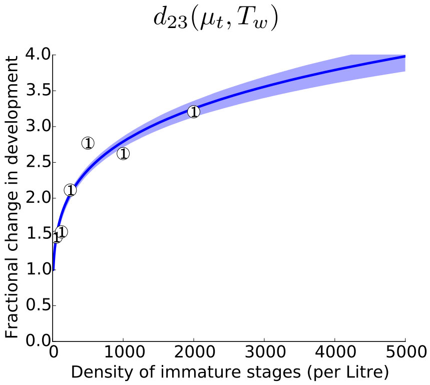<br>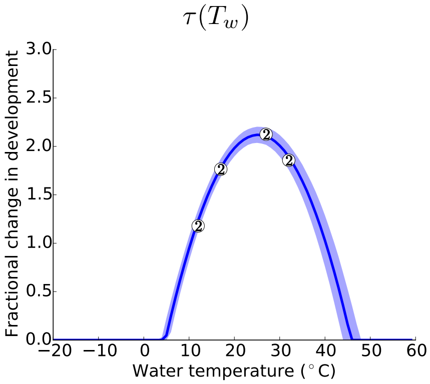	1: [40]<br>2: [22]

References

1. Pumpuni C, Knepler J, Jr GC. Influence of temperature and larval nutrition on the diapause inducing photoperiod of Aedes albopictus. Journal of the American Mosquito Control Association. 1992;8(3):223–227.

2. Lacour G, Vernichon F, Cadilhac N, Boyer S, Lagneau C, Hance T. When mothers anticipate: effects of the prediapause stage on embryo development time and of maternal photoperiod on eggs of a temperate and a tropical strains of Aedes albopictus (Diptera: Culicidae). JOURNAL OF INSECT PHYSIOLOGY. 2014 Dec;71:87–96.

3. Thomas SM, Obermayr U, Fischer D, Kreyling J, Beierkuhnlein C. Low-temperature threshold for egg survival of a post-diapause and non-diapause European aedine strain, Aedes albopictus (Diptera: Culicidae). Parasit Vectors. 2012 Jan;5:100.

4. Delatte H, Gimonneau G, Triboire A, Fontenille D. Influence of Temperature on Immature Development, Survival, Longevity, Fecundity, and Gonotrophic Cycles of Aedes albopictus, Vector of Chikungunya and Dengue in the Indian Ocean. Journal of Medical Entomology. 2009;46(1):33–41.

5. Calado DC, Silva MAN. Avaliação da influência da temperatura sobre o desenvolvimento de Aedes albopictus. Rev Saúde Pública. 2002;36(2):173–179.

6. Calado DC, Navarro-Silva MA. Influência da temperatura sobre a longevidade, fecundidade e atividade hematofágica de Aedes (Stegomyia) albopictus Skuse, 1894 (Diptera, Culicidae) sob condições de laboratório. Rev Bras Entomol. 2002;46:93–98.

7. Juliano S, O'Meara G, Morrill J, Cutwa M. Desiccation and thermal tolerance of eggs and the coexistence of competing mosquitoes. Oecologia. 2002;130(3):458–469.

8. Sota T, Mogi M. Interspecific variation in desiccation survival time of Aedes (Stegomyia) mosquito eggs is correlated with habitat and egg size. Oecologia. 1992;90(3):353–358.

9. Gubler DJ. Comparison of reproductive potentials of Aedes (Stegomyia) albopictus Skuse and Aedes (Stegomyia) polynesiensis Marks. Mosquito News. 1970;30(2):201–209 .

10. Islam MS, Dobson SL. Wolbachia Effects on Aedes albopictus (Diptera: Culicidae) Immature Survivorship and Development. Journal of Medical Entomology. 2006;43(4):689–695.

11. Bellini R, Medici A, Calzolari M, Bonilauri P, Cavrini F, Sambri V, et al. Impact of Chikungunya Virus on Aedes albopictus Females and Possibility of Vertical Transmission Using the Actors of the 2007 Outbreak in Italy. PLoS ONE. 2012;7(2):e28360.

12. Aida HN, Dieng H, Ahmad AH, Satho T, Nurita AT, Salmah MRC, et al. The biology and demographic parameters of Aedes albopictus in northern peninsular Malaysia. Asian Pacific Journal of Tropical Biomedicine. 2011;1(6):472–477.

13. Lounibos LP, O'Meara GF, Juliano SA, Nishimura N, Escher RL, Reiskind MH, et al. Differential Survivorship of Invasive Mosquito Species in South Florida Cemeteries: Do Site-Specific Microclimates Explain Patterns of Coexistence and Exclusion? Annals of the Entomological Society of America. 2010;103(5):757–770.

14. Chang LH, Hsu EL, Teng HJ, Ho CM. Differential Survival of Aedes aegypti and Aedes albopictus (Diptera: Culicidae) Larvae Exposed to Low Temperatures in Taiwan. Journal of Medical Entomology. 2007;44(2):205–210.

15. O'Donnell D, Armbruster P. Inbreeding depression affects life-history traits but not infection by Plasmodium gallinaceum in the Asian tiger mosquito, Aedes albopictus. Infection, Genetics and Evolution. 2010;10(5):669–677.

16. Dieng H, Boots M, Mwandawiro C, Satho T, Hasegawa M, Nyambura GJ, et al. Effects of a copepod predator on the survivorship and development of Aedes albopictus (Diptera: Culicidae). The Japan Society of Medical Entomology and Zoology. 2003;54(2):187–192.

17. Monteiro LCC, De Souza JRB, De Albuquerque CMR. Eclosion rate, development and survivorship of Aedes albopictus (Skuse)(Diptera: Culicidae) under different water temperatures. Neotropical Entomology. 2007;36(6):966–971.

18. Dieng H, Mwandawiro C, Boots M, Morales R, Satho T, Tuno N, et al. Leaf litter decay process and the growth performance of Aedes albopictus larvae (Diptera: Culicidae). Journal of vector ecology. 2002;27:31–38.

19. Medici A, Carrieri M, Scholte EJ, Maccagnani B, Dindo ML, Bellini R. Studies on Aedes albopictus Larval Mass-Rearing Optimization. Journal of Economic Entomology. 2011;104(1):266–273.

20. Alto BW, Juliano SA. Temperature effects on the dynamics of Aedes albopictus (Diptera: Culicidae) populations in the laboratory. Journal of medical entomology. 2001;38(4):548.

21. Alto BW, Juliano SA. Precipitation and temperature effects on populations of Aedes albopictus (Diptera: Culicidae): implications for range expansion. Journal of medical entomology. 2001;38(5):646.

22. Briegel H, Timmermann SE. Aedes albopictus (Diptera: Culicidae): Physiological Aspects of Development and Reproduction. Journal of Medical Entomology. 2001;38(4):566–571.

23. Braks MAH, Juliano SA, Lounibos LP. Superior reproductive success on human blood without sugar is not limited to highly anthropophilic mosquito species. Medical and veterinary entomology. 2006;20(1):53–59.

24. Löwenberg Neto P, Navarro-Silva MA. Development, longevity, gonotrophic cycle and oviposition of Aedes albopictus Skuse (Diptera: Culicidae) under cyclic temperatures. Neotropical Entomology. 2004;33(1):29–33.

25. Muturi EJ, Lampman R, Costanzo K, Alto BW. Effect of Temperature and Insecticide Stress on Life-History Traits of Culex restuans and Aedes albopictus (Diptera: Culicidae). Journal of Medical Entomology. 2011;48(2):243–250.

26. Tsuda Y, Takagi M, Suzuki A, Wada Y. A comparative study on life table characteristics of two strains of Aedes albopictus from Japan and Thailand. Tropical Medicine. 1994;36(1).

27. Udaka M. Some ecological notes on Aedes albopictus Skuse in Shikoku, Japan. Japanese journal of entomology. 1959;27(3):202–208.

28. Hien DS. Biology of Aedes aegypti (L. 1762) and Aedes albopictus (Skuse, 1895) (Diptera, Culicidae). II. Effect of certain environmental conditions on the hatching of larvae. Acta Parasitol Pol. 1975;23:537–552.

29. Dieng H, Saifur RGM, Hassan AA, Salmah MRC, Boots M, Satho T, et al. Indoor-Breeding of Aedes albopictus in Northern Peninsular Malaysia and Its Potential Epidemiological Implications. PLoS ONE. 2010;5(7):e11790.

30. Xue RD, Ali A, Barnard DR. Host species diversity and post-blood feeding carbohydrate availability enhance survival of females and fecundity in Aedes albopictus (Diptera: Culicidae). Experimental parasitology. 2008;119(2):225–228.

31. Xue RD, Barnard DR, Ali A. Influence of Multiple Blood Meals on Gonotrophic Dissociation and Fecundity in Aedes albopictus. Journal of the American Mosquito Control Association. 2009;25(4):504–507.

32. Xue RD, Barnard DR, Ali A. Laboratory and field evaluation of insect repellents as oviposition deterrents against the mosquito Aedes albopictus. Medical and Veterinary Entomology. 2001;15(2):126–131.

33. Aida HN, Hassan AA, Nurita AT, Salmah MRC, Norasmah B. Population analysis of Aedes albopictus (Skuse) (Diptera: Culicidae) under uncontrolled laboratory conditions. Tropical biomedicine. 2008;25(2):117–125.

34. Armbruster P, Hutchinson RA. Pupal Mass and Wing Length as Indicators of Fecundity in Aedes albopictus and Aedes geniculatus (Diptera: Culicidae). Journal of Medical Entomology. 2002;39(4):699–704.

35. Carrieri M, Bacchi M, Bellini R, Maini S. On the Competition Occurring Between Aedes albopictus and Culex pipiens (Diptera: Culicidae) in Italy. Environmental Entomology. 2003;32(6):1313–1321.

36. Muturi EJ, Costanzo K, Kesavaraju B, Alto BW. Can Pesticides and Larval Competition Alter Susceptibility of Aedes Mosquitoes (Diptera: Culicidae) to Arbovirus Infection? Journal of Medical Entomology. 2011;48(2):429–436.

37. Hashim NA, Ahmad AH, Rawi CSM, Tahir NA, Basari N. Life tables study of immature Aedes albopictus (Skuse)(Diptera: Culicidae) during the wet and dry seasons in Penang, Malaysia. The Southeast Asian Journal of Tropical Medicine and Public Health. 2008;.

38. Lounibos LP, Suárez S, Menéndez Z, Nishimura N, Escher RL, O Connell SM, et al. Does temperature affect the outcome of larval competition between Aedes aegypti and Aedes albopictus? Journal of Vector Ecology. 2002;27:86–95.

39. Braks MAH, Honório NA, Lounibos LP, Lourenço-de Oliveira R, Juliano SA. Interspecific Competition Between Two Invasive Species of Container Mosquitoes, Aedes aegypti and Aedes albopictus (Diptera: Culicidae), in Brazil. Annals of the Entomological Society of America. 2004;97(1):130–139.

40. Gavotte L, Mercer DR, Vandyke R, Mains JW, Dobson SL. Wolbachia infection and resource competition effects on immature Aedes albopictus (Diptera: Culicidae). Journal of medical entomology. 2009;46(3):451.

41. Tseng S, Wu I. An ecological study of mosquitoes in Wuhan area. Bulletin of Entomological Research. 1951;42(03):527–533.

42. Liu T. Observations on the physiological age of Culex pipiens pallens Coquillett and Aedes albopictus Skuse. Acta Entomologica Sinica. 1965;14:179–185.

43. Galliard H. Findings on the biology of Culicidae in Hanoi (Tonkin, North Vietnam). I. Comparative larval development of various strains of Aedes albopictus, Aedes aegypti, Culex fatigans & Armigeres obturans. Ann Parasitol Hum Comp. 1958;33:131–144.

44. Halcrow JG. Notes on a laboratory colony of Aedes (Stegomyia) albopictus (Skuse) and its distribution in Mauritius. Proceedings of the Royal Entomological Society of London. 1955;30:40–42.

45. Hien DS. Biology of Aedes aegypti (L. 1762) and Aedes albopictus (Skuse, 1895) (Diptera, Culicidae). III. Effect of certain environmental conditons on the development of larvae and pupae. Acta Parasitol Pol. 1975;23:553–568.
