# Supplementary figures and images for: Large-Scale Modelling of the Environmentally-Driven Population Dynamics of Temperate Aedes albopictus (Skuse)
Source: PLoS One. 2016 Feb 12;11(2):e0149282. doi: 10.1371/journal.pone.0149282 (PMC4752251; doi:10.1371/journal.pone.0149282)

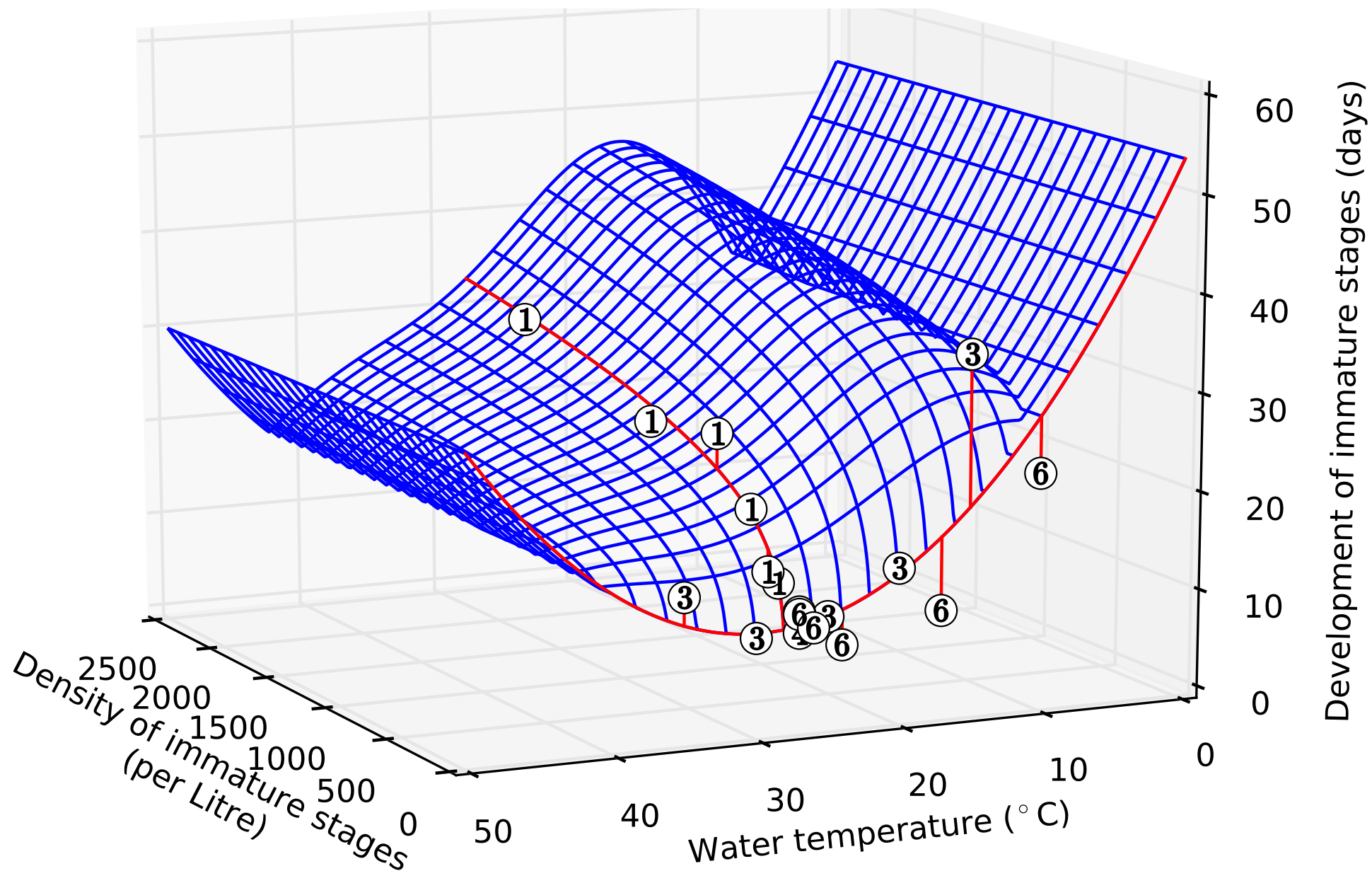

Figure S.1. Temperature- and density-dependent development of the immature stages.

Supplement: S1 Fig — The blue surface indicates the expected duration of larva and pupa, (d2+d3)d23, with respect to water temperature, Tw, and density of the immature stages, μ. Observed duration of the immature stages are given as white circles and the difference between observed and expected values are marked with horizontal red lines. 1: Gavotte et al. 2009, 3: Delatte et al. 2009, 4: Liu 1965, 5: Halcrow 1955, 6: Udaka 1959 (see S1 Table for the references). (PDF) [file pone.0149282.s005.pdf]

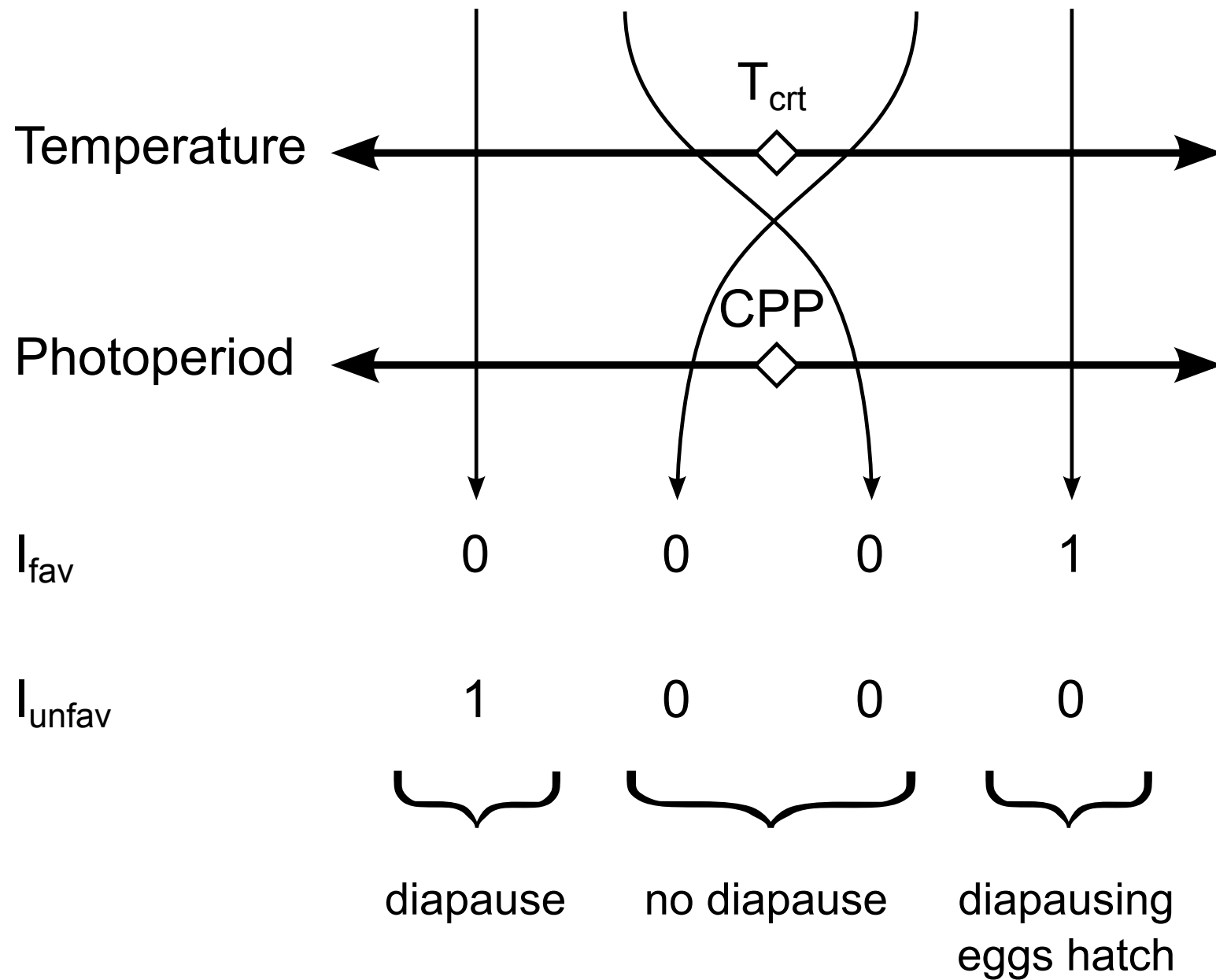

Figure S.2. Decision making for the diapausing behaviour.

Supplement: S2 Fig — The diagram outlines the algorithm with which the indicator functions, Ifav and Iunfav, determine suitable conditions for diapausing and egg hatching with respect to temperature and photoperiod. (PDF) [file pone.0149282.s006.pdf]

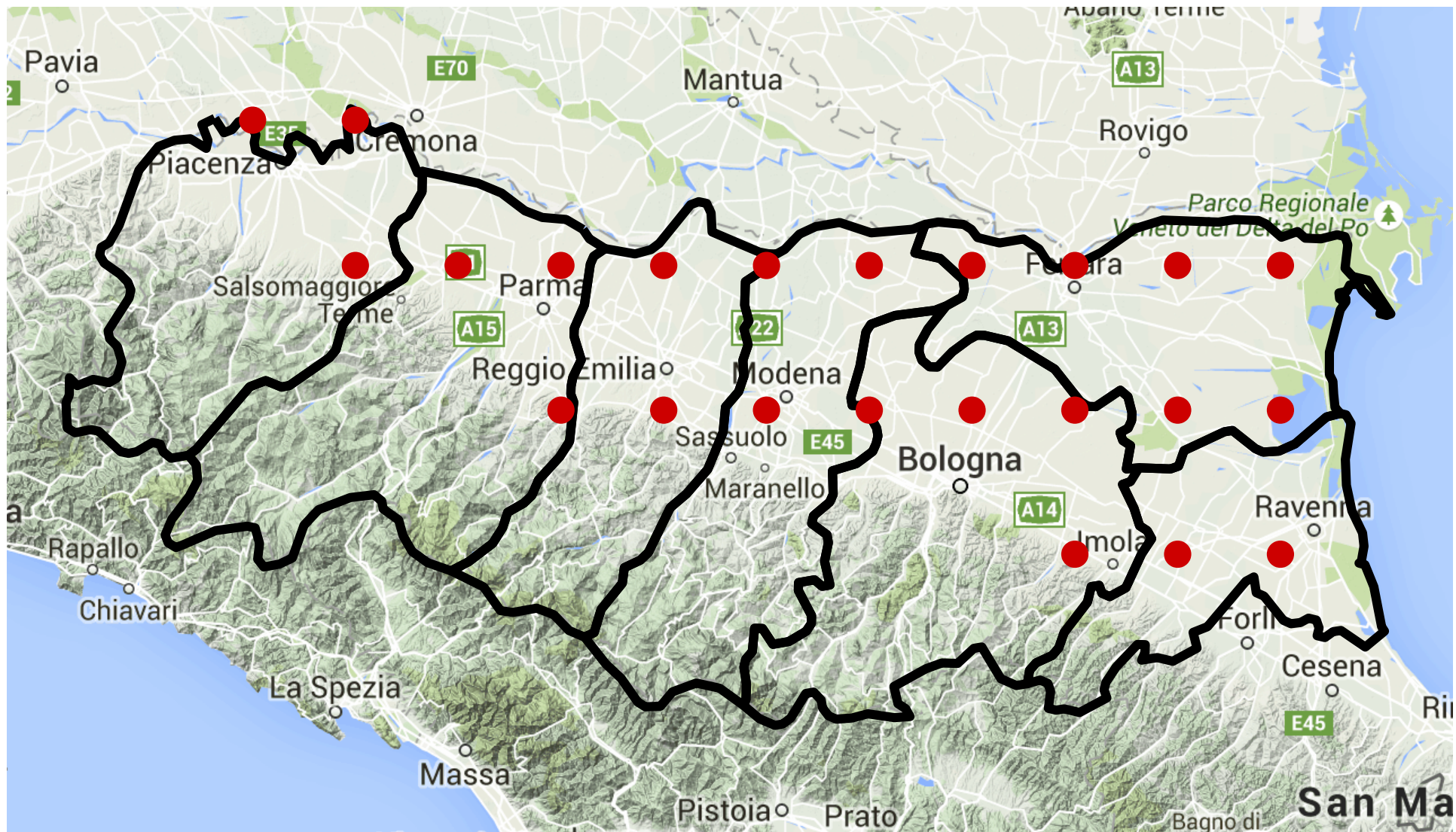

Figure S.3. Grid points of the higher-resolution datasets used for parameter inference.

Supplement: S3 Fig — (PDF) [file pone.0149282.s007.pdf]

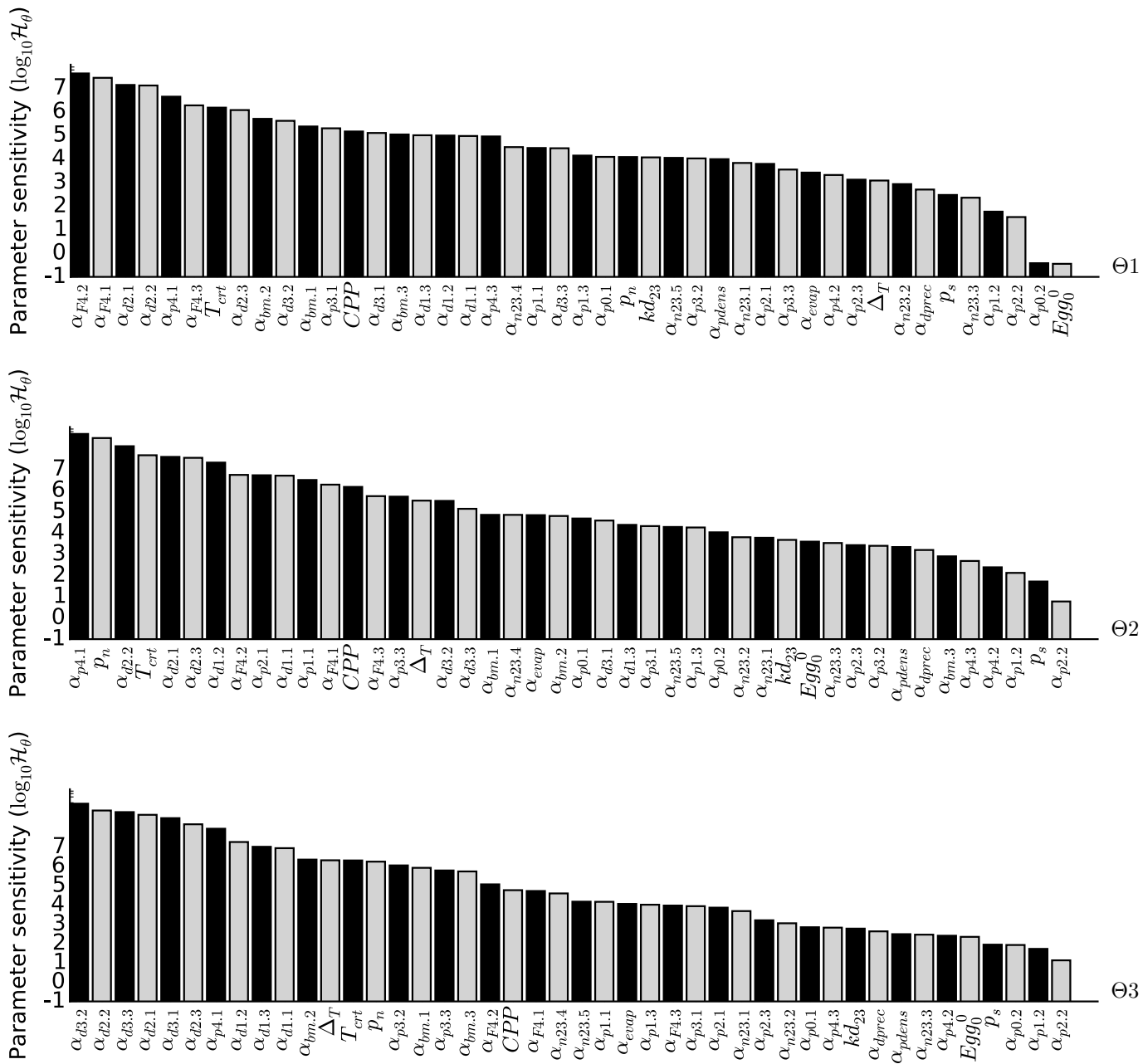

Figure S.5. Sensitivity analysis for  $\Theta_1$ ,  $\Theta_2$  and  $\Theta_3$ .

Supplement: S5 Fig — (PDF) [file pone.0149282.s009.pdf]

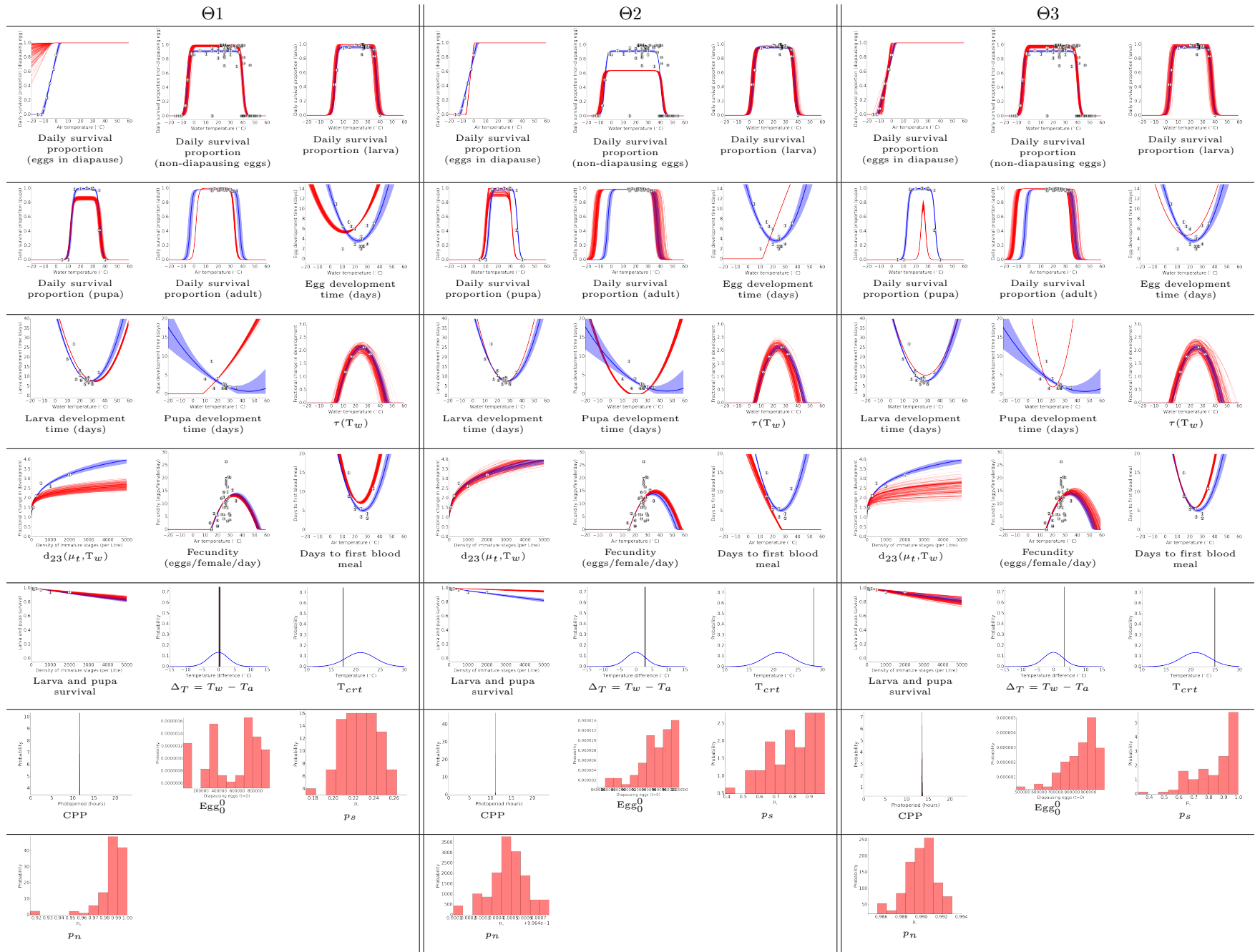

Figure S.6. Comparison of prior and posterior distributions  $\Theta_1$ ,  $\Theta_2$  and  $\Theta_3$ .

Supplement: S6 Fig — The prior distribution is represented by a solid line (mean) and a shaded region (95% confidence interval). The posterior distribution is represented by 100 samples drawn from each posterior mode. Data from literature are plotted as numbered circles (see S1 Table for the references). Figures are grouped in three columns, one for each posterior mode, Θ1, Θ2 and Θ3. (PDF) [file pone.0149282.s010.pdf]
