# Supplementary material for: Large-Scale Modelling of the Environmentally-Driven Population Dynamics of Temperate Aedes albopictus (Skuse)
Source: PLoS One. 2016 Feb 12;11(2):e0149282. doi: 10.1371/journal.pone.0149282 (PMC4752251; doi:10.1371/journal.pone.0149282)
Supplement: S4 Fig — (PDF) [file pone.0149282.s008.pdf]

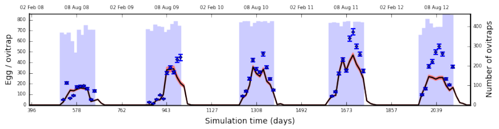
Bologna (Θ1)

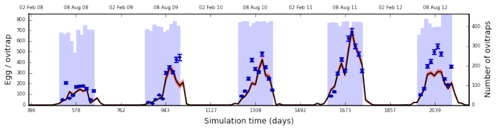
Bologna (Θ2)

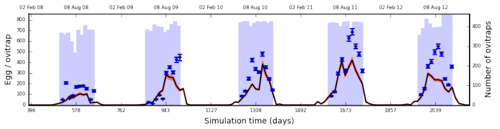
Bologna (Θ3)

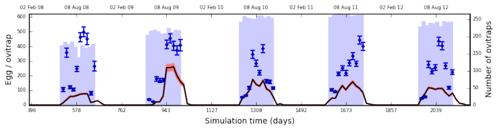
Ferrara (Θ1)

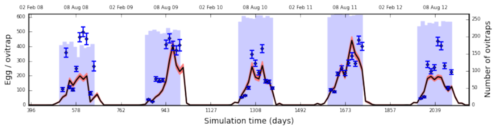
Ferrara (Θ2)

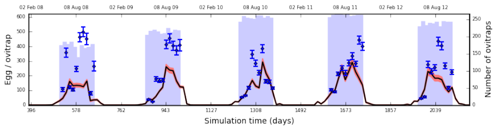
Ferrara (Θ3)

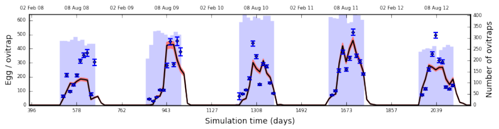
Modena (Θ1)

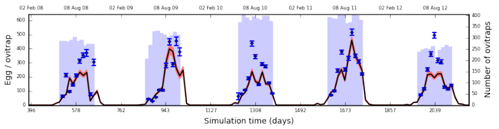
Modena (Θ2)

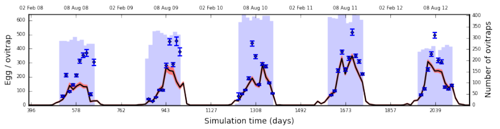
Modena (Θ3)

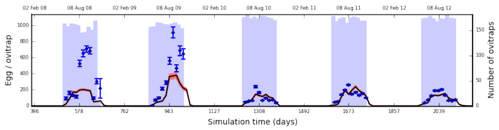
Piacenza (Θ1)

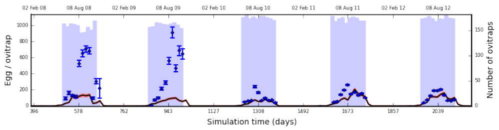
Piacenza (Θ2)

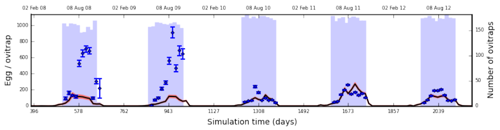
Piacenza (Θ3)

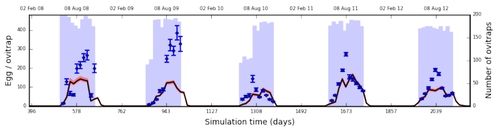
Parma (Θ1)

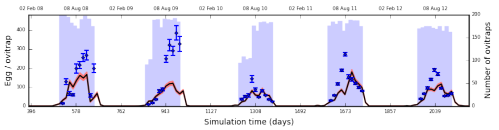
Parma (Θ2)

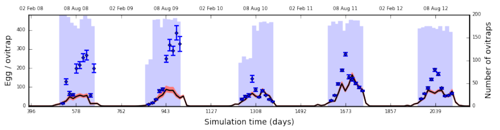
Parma (Θ3)

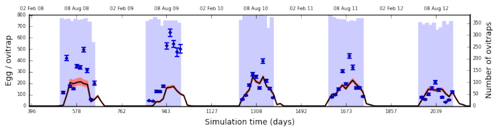
Ravenna (Θ1)

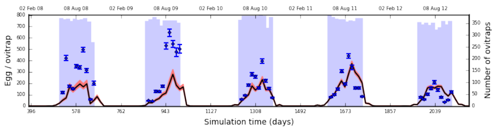
Ravenna (Θ2)

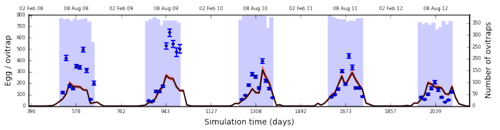
Ravenna (Θ3)

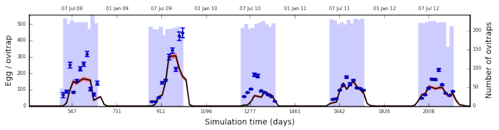
Reggio Emilia (Θ1)

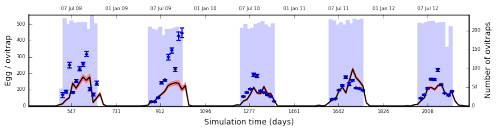
Reggio Emilia (Θ2)

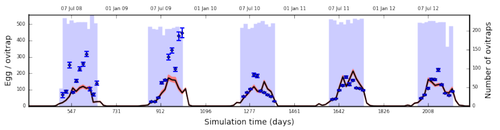
Reggio Emilia (Θ3)

Figure S.4. Evaluating model performance over Emilia-Romagna with Θ1, Θ2 and Θ3.
