# Supplementary material for: Large-Scale Modelling of the Environmentally-Driven Population Dynamics of Temperate Aedes albopictus (Skuse)
Source: PLoS One. 2016 Feb 12;11(2):e0149282. doi: 10.1371/journal.pone.0149282 (PMC4752251; doi:10.1371/journal.pone.0149282)
Supplement: S7 Fig — Solid black line shows the mean while red shade indicates the 95% confidence interval. Figures are grouped in three columns, one for each posterior mode, Θ1, Θ2 and Θ3. (PDF) [file pone.0149282.s011.pdf]

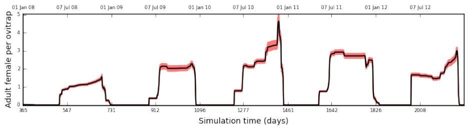

Bologna (Θ1)

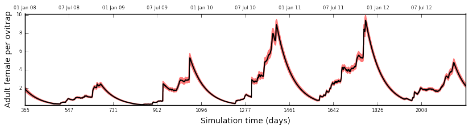

Bologna (Θ2)

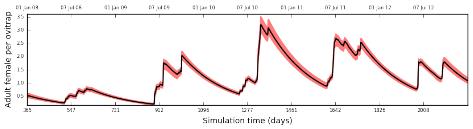

Bologna (Θ3)

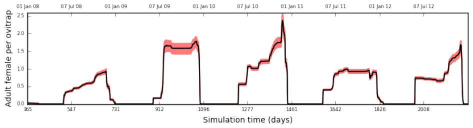

Ferrara (Θ1)

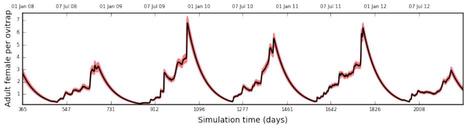

Ferrara (Θ2)

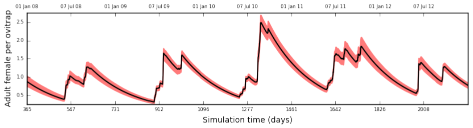

Ferrara (Θ3)

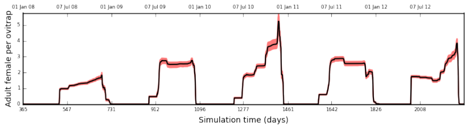

Modena (Θ1)

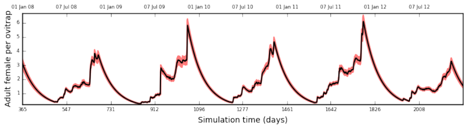

Modena (Θ2)

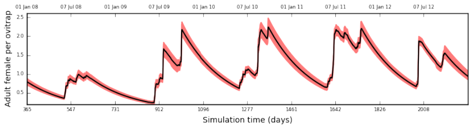

Modena (Θ3)

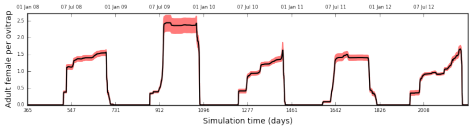

Piacenza (Θ1)

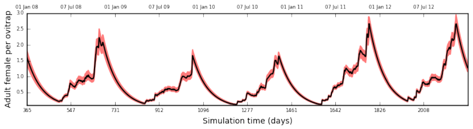

Piacenza (Θ2)

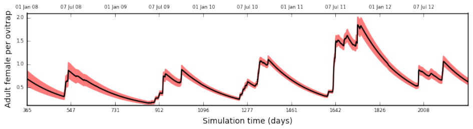

Piacenza (Θ3)

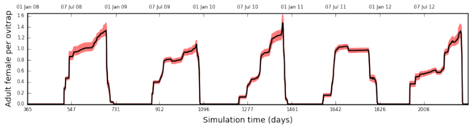

Parma (Θ1)

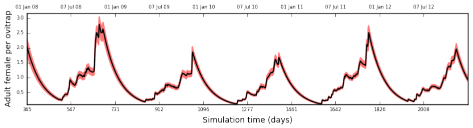

Parma (Θ2)

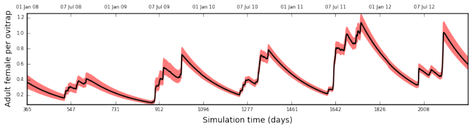

Parma (Θ3)

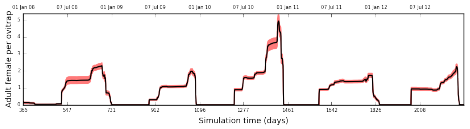

Ravenna (Θ1)

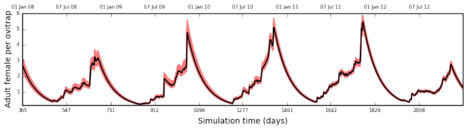

Ravenna (Θ2)

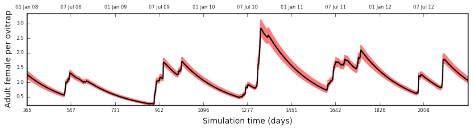

Ravenna (Θ3)

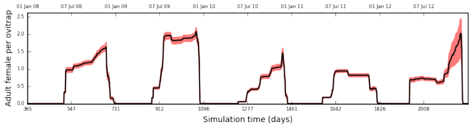

Reggio Emilia (Θ1)

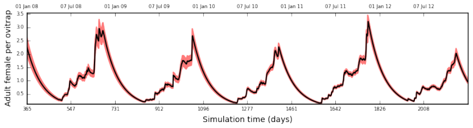

Reggio Emilia (Θ2)

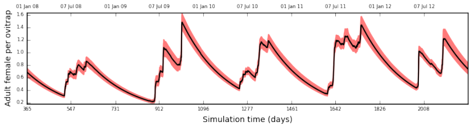

Reggio Emilia (Θ3)

Figure S.7. Simulated number of adult females per ovitrap.
