# Supplementary material for: Large-Scale Modelling of the Environmentally-Driven Population Dynamics of Temperate Aedes albopictus (Skuse)
Source: PLoS One. 2016 Feb 12;11(2):e0149282. doi: 10.1371/journal.pone.0149282 (PMC4752251; doi:10.1371/journal.pone.0149282)
Supplement: S9 Fig — Solid black line shows the mean while red shade indicates the 95% confidence interval. Figures are grouped in three columns, one for each posterior mode, Θ1, Θ2 and Θ3. (PDF) [file pone.0149282.s013.pdf]

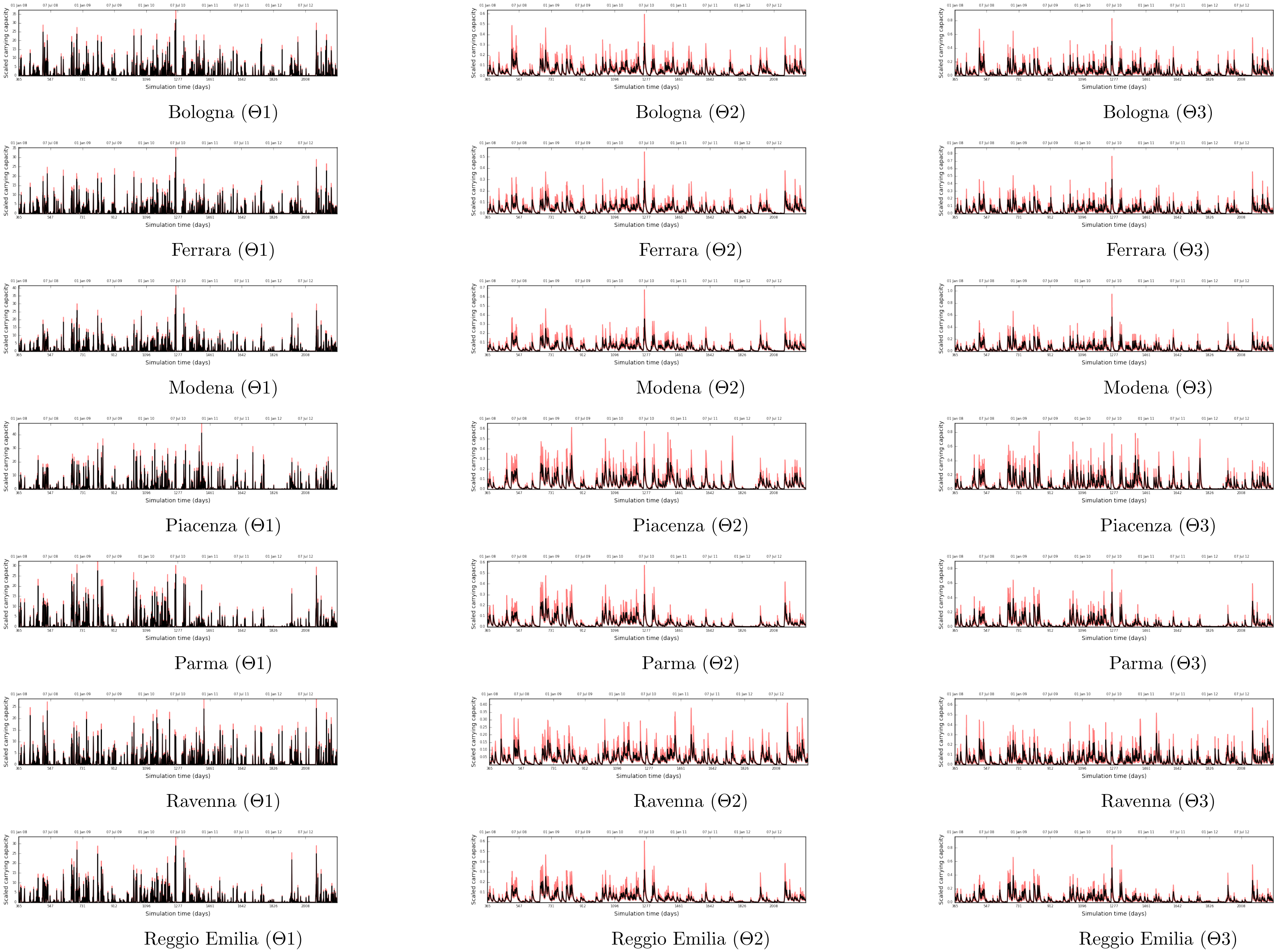

Figure S.9. Simulated scaled carrying capacity, $\mathcal{K}$, per ovitrap.
