# Supplementary material for: Large-Scale Modelling of the Environmentally-Driven Population Dynamics of Temperate Aedes albopictus (Skuse)
Source: PLoS One. 2016 Feb 12;11(2):e0149282. doi: 10.1371/journal.pone.0149282 (PMC4752251; doi:10.1371/journal.pone.0149282)
Supplement: S10 Fig — (PDF) [file pone.0149282.s014.pdf]

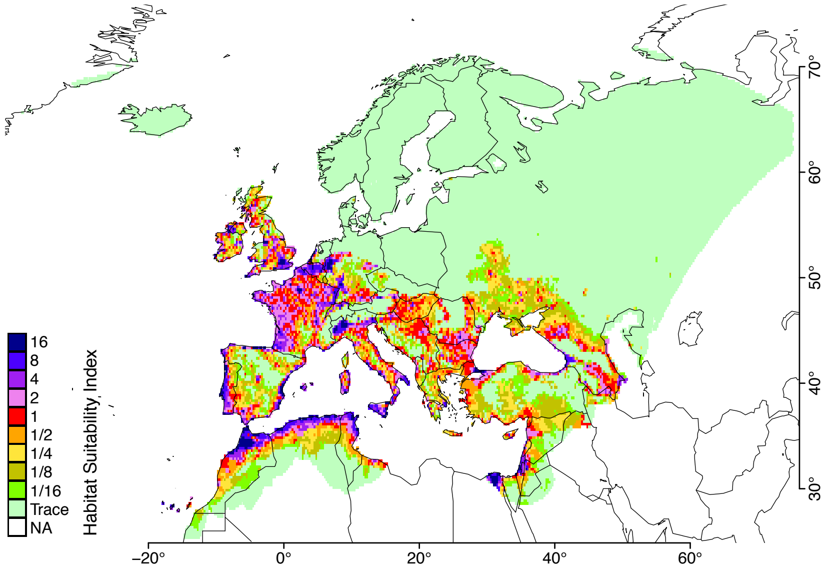

$\Theta 1$

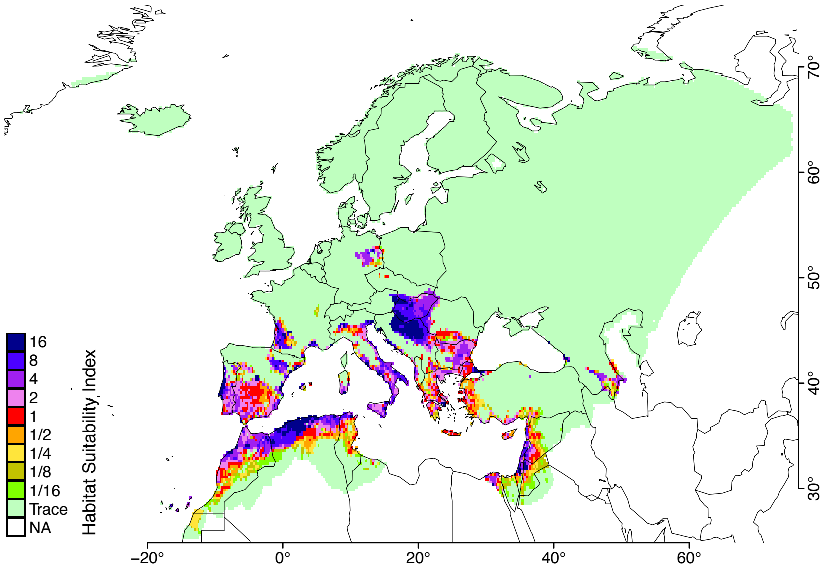

$\Theta 2$

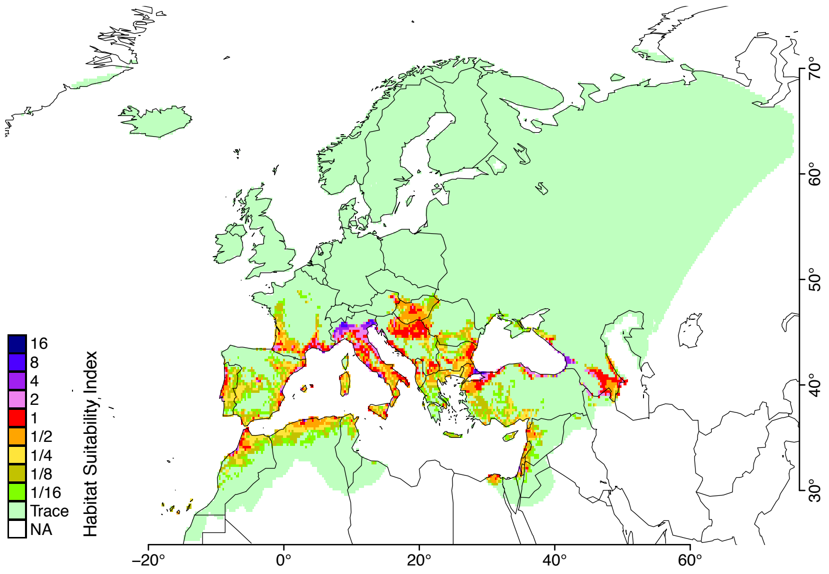

$\Theta 3$

Figure S.10. Habitat suitability indices (HSI) for Europe with $\Theta 1$, $\Theta 2$ and $\Theta 3$.
